# Diversity of myxozoans (Cnidaria) infecting Neotropical fishes in southern Mexico

**DOI:** 10.1038/s41598-023-38482-2

**Published:** 2023-07-26

**Authors:** Gema Alama-Bermejo, Jesús S. Hernández-Orts, Martín García-Varela, Alejandro Oceguera-Figueroa, Hana Pecková, Ivan Fiala

**Affiliations:** 1 Division of Fish Health, University of Veterinary Medicine, Veterinärplatz 1, 1210 Vienna, Austria; 2 Natural History Museum, London, Cromwell Road, SW7 5BD London, United Kingdom; 3 Institute of Parasitology, Biology Centre, Czech Academy of Sciences, Branišovská 31, 370 05 České Budějovice, Czech Republic; 4 Departamento de Zoología, Instituto de Biología, Universidad Nacional Autónoma de México, Ciudad Universitaria, 04510 Mexico City, Mexico

**Keywords:** Parasitology, Biodiversity

## Abstract

Myxozoans are a unique group of microscopic parasites that infect mainly fishes. These extremely reduced cnidarians are highly diverse and globally distributed in freshwater and marine habitats. Myxozoan diversity dimension is unknown in Mexico, a territory of an extraordinary biological diversity. This study aimed to explore, for the first time, myxozoan parasite diversity from fishes of the Neotropical region of Mexico. We performed a large morphological and molecular screening using host tissues of 22 ornamental and food fish species captured from different localities of Veracruz, Oaxaca and Chiapas. Myxozoan infections were detected in 90% of the fish species, 65% of them had 1 or 2 and 35% had 3 and up to 8 myxozoan species. Forty-one putative new species were identified using SSU rDNA phylogenetic analyses, belonging to two main lineages: polychaete-infecting (5 species) and oligochaete-infecting (36 species) myxozoans; from those we describe 4 new species: *Myxidium zapotecus* sp. n., *Zschokkella guelaguetza* sp. n., *Ellipsomyxa papantla* sp. n. and *Myxobolus zoqueus* sp. n. Myxozoan detection increased up to 6 × using molecular screening, which represents 3.7 × more species detected than by microscopy. This study demonstrated that Neotropical fishes from Mexico are hosts of a multitude of myxozoans, representing a source of emerging diseases with large implications for economic and conservation reasons.

## Introduction

Myxozoans are microscopic cnidarian parasites that are globally distributed in marine and freshwater habitats. These spore-forming parasites are strongly reduced in size and morphology when compared to their free-living cnidarian relatives. Myxozoans have complex life cycles that involved annelids and bryozoans as definitive invertebrate hosts and predominantly fishes as intermediate vertebrate hosts. In both hosts, waterborne, infectious spores are formed; actinospores in the invertebrate host and myxospores in the vertebrate host. These parasites are particularly well known for the disease they cause in aquaculture and wild fish stocks^1^.

Myxozoans are a highly diverse group, with > 2400 species, representing approximately one fifth of the phylum Cnidaria^2,3^. However, the biodiversity of myxozoan species remains unknown and is very likely underestimated^4^, especially in certain geographic areas. Southern Mexico is included in the so-called Mesoamerican biodiversity hotspot, which includes areas that have high concentrations of endemic species and that are experiencing exceptional loss of habitat^5^. Myxozoans are considered an understudied group in Mexico, especially when compared to other parasitic groups with long tradition in the Mexican fish parasitology research^6,7^. So far, only six species of myxozoans have been reported in Mexico, three from marine habitats, i.e. *Kudoa dianae* Dyková, Fajer Avila & Fiala, 2002, *Myxobolus mexicanus* Yoshino & Noble, 1973 and *Myxidium coryphaenoidium* Noble, 1966, and three from freshwater habitats i.e. *Myxobolus nuevoleonensis* Salinas, Jiménez-Guzmán, Galaviz-Silva & Ramírez-Bon, 1991, *Myxobolus cartilaginis* Hoffman, Putz & Dunbar, 1965 and *Henneguya exilis* Kudo, 1929^8–14^. From these, only *K. dianae* and *M. coryphaenoidium* have molecular data available^13,15^. Additionally, a myxozoan infection episode in tilapia was reported in Mexico, without species identification of the causative agent^16^.

During wild fish collection campaigns in the neotropical region of Mexico, myxozoan infections were detected. This study seeks to explore myxozoan species diversity from fishes in southern Mexico, using morphological and molecular tools. We provide the morphological and molecular characterization of four new myxozoan species, as well as their phylogenetic relationships. Using host tissues, hidden diversity and occurrence of mixed infections were further investigated through a large-scale molecular screening using PCR techniques and Sanger sequencing. This is the first comprehensive study on the diversity of this group of parasitic cnidarians in Mexico.

## Materials and methods

### Fishes and samples collection

**Figure 1:**
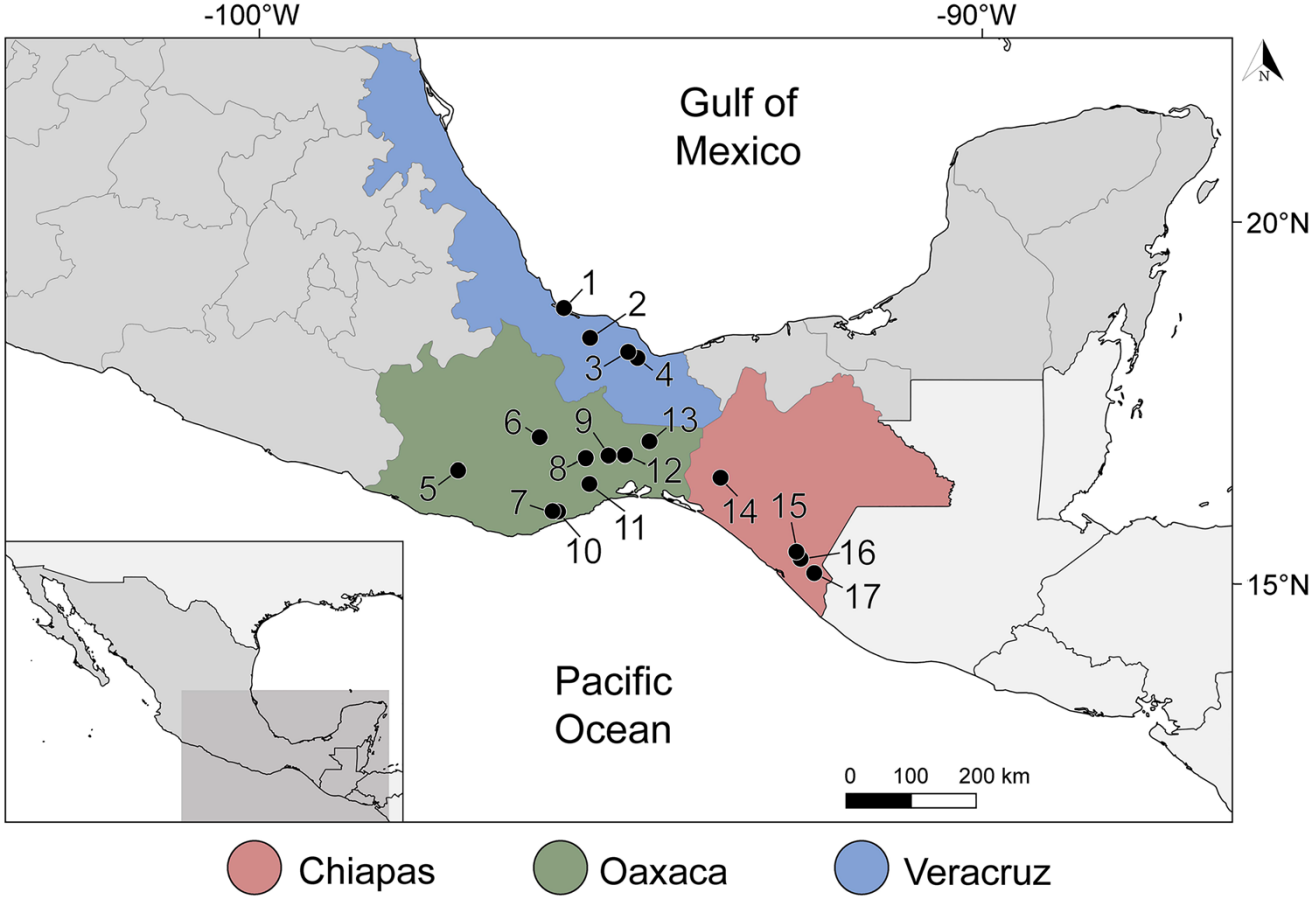
Sampling localities of freshwater and marine fishes along the Neotropical Region of Mexico. Coordinates are provided in Supplementary Data 1. Legend: 1, Alvarado, Veracruz; 2, Tlacotalpan, Veracruz; 3, Catemaco, Veracruz; 4, Río La Palma, Veracruz; 5, El Toronjo, Oaxaca; 6, Río Grande, Mitla, Oaxaca; 7, Río los Sabinos, Oaxaca; 8, Río los Perros, Santa María, Oaxaca; 9, Santa María, Guienagati, Oaxaca; 10, Río Chacalapa, Oaxaca; 11, Río Tequisistlán, Oaxaca; 12, Rio Grande, Matías Romero, Oaxaca; 13, Río Negro, Santa María Chimalapa, Oaxaca; 14, Río San Juan, Cristóbal Colón, Chiapas; 15, Río San Juan, Cristóbal Obregón, Chiapas; 16, Nueva Francia, Chiapas; 17, El Triunfo, Chiapas; 18, Río Huixtla, Chiapas. The map was generated using QGIS v.3.32 (Free and Open Source Geographic Information System, https://www.qgis.org/en/site/#).

A total of 120 fish specimens belonging to 22 species, 10 families and 6 orders, were captured using electrofishing and gillnets from 17 localities in 3 states in southern Mexico: Veracruz, Oaxaca and Chiapas (Fig. 1, Supplementary Data 1) between March 2014 and March 2015. In addition, one Atlantic bonito *Sarda sarda* was purchased in the local market of Alvarado, Veracruz in May 2014. Captured fish were immediately transported to the field laboratory in tanks containing aerated water from the original locality. Fish were euthanized by neural pithing, dissected and species were identified following^17,18^. Gall bladder was punctured, and bile collected in a tube. Fresh bile smears were examined under the microscope and digital images of the myxospores obtained from fresh material during fieldwork when possible. Aliquots of each bile sample were fixed for molecular (99% ethanol) and morphological analyses (10% neutral buffered formalin). Additionally, pieces of intestine and kidney of nine fish specimens (Intestine and kidney: *Mayaheros urophthalmus* (n = 2), *Parachromis friedrichsthalii* (n = 1), *Vieja fenestrata* (n = 1), *Rhamdia guatemalensis* (n = 3); only intestine—*Poecilia sphenops* (n = 1), *Dormitator maculatus* (n = 1); only kidney—*Dajaus monticola* (n = 1), *Paraneetroplus bulleri* (n = 1); Table 1) were examined under the microscope and preserved in absolute ethanol for molecular screening. In order to avoid DNA cross contamination, scissors and tweezers were flame sterilized during sampling. Fish names, habitats and human uses follow FishBase^19^. The sampling in this work complies with the current laws and animal ethics regulations of Mexico. Fishes were collected under the Cartilla Nacional de Colector Científico (FAUT 0202) issued by the Secretaría del Medio Ambiente y Recursos Naturales (SEMARNAT), to M.G.V.

**Table 1:** Total prevalence of myxozoans detected by light microscopy and by PCR screening (SSUrDNA) of gall bladder, intestine (*) and kidney ( +) of fish hosts (n = 120) analyzed between 2014 and 2015 from different localities in Oaxaca, Chiapas and Veracruz states, Mexico.

Fish species	Locality, number of fish and year	Myxozoan prevalence				# Sequences
		Microscopy	Total microscopy	PCR	Total PCR	
*Profundulus punctatus* (Günther (n = 23)	Río San Juan, Cristobal Obregón, Chiapas (n = 2) 2014	0/2	22,2% (4/18)	0/2	45,5% (10/22)	–
	Río Huixtla, Chiapas (n = 3) 2014	0/1		2/3		2
	Nueva Francia, Chiapas (n = 1) 2014	–		1/1		1
	El Triunfo, Chiapas (n = 5) 2014	0/3		3/5		3
	Río los Perros, Santa María, Oaxaca (n = 7) 2015	4/7		4/7		4
	Río Chacalapa, Oaxaca (n = 5) 2015	0/5		0/4		–
*Profundulus oaxacae* (Meek) (n = 14)	Río Los Sabinos, Oaxaca (n = 7) 2015	1/7	14,3% (2/14)	3/6	25% (3/12)	3
	El Toronjo, Oaxaca (n = 5) 2015	0/5		0/5		–
	Río Grande, Mitla, Oaxaca (n = 2) 2015	1/2		0/1		–
*Tlaloc labialis* (Günther) (n = 1)	Río San Juan, Cristobal Obregón, Chiapas (n = 1) 2014	0/1	0/1	0/1	0/1	–
*Poecilia mexicana* Steindachner (n = 8)	Río La Palma, Veracruz (n = 7) 2014	0/7	0/7	2/5	50% (3/6)	4
	Tlacotalpan, Veracruz (n = 1) 2014	–		1/1		1
*Poecilia sphenops* Valenciennes (n = 6)	Tlacotalpan, Veracruz (n = 5) 2014	–	0/1	1/1 ^(^*^)^	0/1, 1/1^(^*^)^	1
	Santa Maria, Guienagati, Oaxaca (n = 1) 2015	0/1		0/1		–
*Xiphophorus alvarezi* Rosen (n = 4)	Río La Palma, Veracruz (n = 4) 2014	0/4	0/4	2/2	2/2	4
*Dajaus monticola* (Bancroft) (n = 7)	Río La Palma, Veracruz (n = 4) 2014	0/4	14,3% (1/7)	4/4	85,7% (6/7); 0/1^(+)^	7
	Rio Grande, Matías Romero, Oaxaca (n = 3) 2015	1/3		2/3; 0/1^(+)^		3
*Mayaheros urophthalmus* (Günther) (n = 4)	Tlacotalpan, Veracruz (n = 2) 2014	0/2	0/4; 0/2; 0/2	1/1	66,7% (2/3); 0/2^(^*^)^; 0/2^(+)^	1
	Tlacotalpan, Veracruz (n = 2) 2015	0/2;0/2^(^*^)^;0/2^(+)^		1/2;0/2^(^*^)^;0/2^(+)^		2
*Parachromis friedrichsthalii* (Heckel) (n = 4)	Río Grande, Matías Romero, Oaxaca (n = 4) 2015	1/4	25% (1/4)	1/4; 0/1^(^*^)^; 0/1^(+)^	25% (1/4); 0/1^(^*^)^; 0/1^(+)^	1
*Paraneetroplus bulleri* Regan (n = 3)	Río Negro, Santa María Chimalapa, Oaxaca (n = 2) 2014	0/2	0/3	1/1	1/2;0/1^(+)^	1
	Río Grande, Matías Romero, Oaxaca (n = 1) 2015	0/1		0/1;0/1^(+)^		–
*Thorichthys maculipinnis* (Steindachner) (n = 2)	Río Grande, Matías Romero, Oaxaca (n = 2) 2015	1/2	1/2	1/2	1/2	1
*Vieja fenestrata* (Günther) (n = 2)	Río Grande, Matías Romero, Oaxaca (n = 2) 2015	1/2	1/2	1/2; 0/1^(^*^)^; 0/1^(+)^	1/2; 0/1^(^*^)^; 0/1^(+)^	2
*Vieja zonata* (Meek) (n = 1)	Río Tequisistlán, Oaxaca (n = 1) 2015	0/1	0/1	1/1	1/1	1
*Cichlasoma trimaculatum* (Günther) (n = 1)	Río Tequisistlán, Oaxaca (n = 1) 2015	0/1	0/1	0/1	0/1	–
*Maskaheros regani* (Miller) (n = 1)	Río Grande, Matías Romero, Oaxaca (n = 1) 2015	0/1	0/1	1/1	1/1	1
*Dormitator maculatus* (Bloch) (n = 11)	Tlacotalpan, Veracruz (n = 6) 2014	2/6	27,3% (3/11)	3/6	45.5% (5/11); 1/1^(^*^)^	6
	Tlacotalpan, Veracruz (n = 5) 2015	1/5		2/5;1/1^(^*^)^		4
*Gobiomorus dormitor* Lacepède (n = 2)	Río La Palma, Veracruz (n = 2) 2014	0/2	0/2	1/2	1/2	1
*Awaous banana* (Valenciennes) (n = 3)	Río Negro, Santa María Chimalapa, Oaxaca (n = 1) 2014	1/1	100% (3/3)	1/1	100% (3/3)	1
	Río Grande, Matías Romero, Oaxaca (n = 2) 2015	2/2		2/2		3
*Sarda sarda* (Bloch) (n = 1)	Alvarado, Veracruz (n = 1) 2014	0/1	0/1	1/1	1/1	4
*Rhamdia quelen* (Quoy & Gaimard) (n = 6)	Río Negro, Santa María Chimalapa, Oaxaca (n = 1) 2014	0/1	0/4	1/1	33,3% (2/6)	1
	Río San Juan, Cristobal Obregón, Chiapas (n = 5) 2014	0/3		1/5		1
*Rhamdia guatemalensis* (Günther) (n = 4)	Río Grande, Matías Romero, Oaxaca (n = 2) 2015	0/2;0/1^(^*^)^	0/4; 0/2; 0/1	0/2; 0/2^(^*^)^;0/2^(+)^	0/3;1/3^(^*^)^;1/3^(+)^	–
	Santa María, Guienagati, Oaxaca (n = 1) 2015	0/1		–		–
	Catemaco, Veracruz (n = 1) 2015	0/1;0/1^(^*^)^;0/1^(+)^		0/1;1/1^(^*^)^;1/1^(+)^		2
*Synbranchus marmoratus* Bloch (n = 2)	Río Negro, Santa María Chimalapa, Oaxaca (n = 1) 2014	0/1	0/2	1/1	1/2	2
	Río Huixtla, Chiapas (n = 1) 2014	0/1		0/1		–
*Xiphophorus* sp.(n = 1)	Río La Palma, Veracruz (n = 1) 2014	0/1	0/1	1/1	1/1	1
*Astyanax* sp. (n = 3)	Río Negro, Santa María Chimalapa, Oaxaca (n = 3) 2014	0/3	0/3	1/2	1/2	3
*Eleotris* sp. (n = 2)	Río La Palma, Veracruz (n = 2) 2014	0/2	0/2	1/1	1/1	1
*Paraneetroplus* sp. (n = 2)	Río Negro, Santa María Chimalapa, Oaxaca (n = 2) 2014	1/2	1/2	2/2	2/2	6
*Thorichthys* sp. (n = 2)	Río Negro, Santa María Chimalapa, Oaxaca (n = 2) 2014	0/1	0/1	0/1	0/1	–
Total	n = 120		16% (17/106)		49% (50/102); 33.3% (3/9)^(*)^; 11.1% (1/9) ^(+)^	79

### Morphological analyses

Digital images of plasmodia and myxospores were taken using a Leica DM750 microscope and Leica ICC50 HD digital camera (1000×) for fresh material during field work; an Olympus BX51 microscope and an Olympus DP70 digital camera (1000×) was used for formalin-fixed material at the lab. Myxospores measurements (n = 162) followed recommendations of^20^ but using the term polar tubule instead of polar filament (see^21^). Measurements were taken from digital images using ImageJ 1.47v^22^ and are given in µm as the mean ± standard deviation with the range in parentheses. Myxospores were air dried directly onto glass slides, stained with Epredia™ Shandon™ Kwik-Diff™ Stains (Thermo Fisher Scientific; Waltham, Massachusetts) and mounted with Neo-mount™ non-aqueous mounting medium (Merck; Darmstadt, Germany). Archival smears are deposited at the Institute of Biology (Universidad Nacional Autónoma de México, UNAM), Colección Nacional de Helmintos CNHE 11950-11953.

Two bile samples were fixed in 2.5% glutaraldehyde in 0.1M PBS and processed for scanning electron microscopy (SEM), by adhering the myxospores to a poly-D-lysine coated coverslip, followed by postfixation and dehydratation according to^23^. Critical point drying and gold sputtered-coating was performed at the Laboratory of Electron Microscopy, Institute of Parasitology, Czech Academy of Sciences, Czech Republic. Samples were examined using a JEOL JSM-7401 F (JEOL Ltd., Japan) electron microscope.

### Molecular analyses

Bile (n = 102), intestine (n = 9) and kidney (n = 9) samples were used for total DNA extraction. Bile content was centrifuged for 5 min at 14.000 RPM to form a pellet and ethanol was removed. Pellet/tissue was airdried until complete ethanol evaporation and resuspended in TNES -Urea (10 mM Tris–HCl (pH 8), 125 mM NaCl, 10 mM EDTA, 0.5% SDS, 4M urea), digested with 100 µg/ml of proteinase K, overnight at 55 °C, and extracted following a standard phenol–chloroform protocol^24^. The extracted DNA was re-suspended in 30–100 µL RNAse/DNAse-free water. Small subunit ribosomal SSU rDNA amplicons were first amplified using primers ERIB1 (5′-ACC TGG TTG ATC CTG CCA G-3′) and ERIB10 (5′-CTT CCG CAG GTT CAC CTA CGG-3')^25^ followed by a nested PCR with Myxgp2f (5′-WTG GAT AAC CGT GGG AAA-3′)^26^ and ACT1r (5′-AAT TTC ACC TCT CGC TGC CA-3′)^27^. This PCR protocol was used to screen all samples obtained in this study. PCRs were conducted in 10 μl reactions with 0.025 Uμl^−1^ TITANIUM Taq DNA polymerase and 10 × buffer which contained 1.5 mM MgCl_2_ (BD Biosciences Clontech), with 0.2 mM of each dNTP, 0.5 µM of each primer, and 10–150 ng of template DNA or 0.5 µL of PCR product. Cycling conditions of initial PCR consisted of 95 °C for 2 min, followed by 35 cycles of 95 °C for 50 s, 60ºC for 50 s and 68 °C for 2 min, and final extension 68 °C for 4 min. Nested PCR consisted of 95 °C for 3 min, followed by 30 cycles of 95 °C for 50 s, 58 °C for 50 s and 68 °C for 1 min 20 s with final extension 68 °C for 4 min. Expected DNA amplicons (≈900–1000 bp) were visualized in 1% agarose gel in sodium acetate buffer and purified for sequencing using a Gel/PCR DNA Fragments Extraction Kit (Geneaid Biotech Ltd., USA). Preferably, direct sequencing of PCR products using nested PCR primers was attempted. Additional primers were used to obtain longer fragments of the SSU rDNA region for some myxospore positive samples (Supplementary Data 2). Problematic amplicons (double peaks, poor quality sequences) were cloned into the pDrive Cloning vector (Qiagen PCR Cloning Kit, Germany) and transformed into the competent *E. coli* strain XL-1. Plasmid DNA was isolated using a High Pure Plasmid Isolation Kit (Roche Applied Science, Germany) and sequenced using primers M13F (5′-GTA AAA CGA CGG CCA G-3′) and M13R (5´- AAC AGC TAT GAC CAT G -3´). Sequences were obtained with an ABI PRISM 3130 × 1 automatic sequencer (Applied Biosystems; Prague, Czech Republic). The overlapping partial sequences of SSU rDNA were trimmed and assembled into single consensus contigs using Geneious Prime 2022.2 (Biomatters; Auckland, New Zealand; https://www.geneious.com) and submitted to GenBank (accession numbers OQ888222-OQ888300; see Supplementary Data 3).

### Phylogenetic analyses

To determine the phylogenetic relationships of identified myxozoans, two sequence alignments were performed, one for myxozoans clustering in the oligochaete-infecting lineage and a second for myxozoans clustering in the polychaete-infecting lineage (see^28^). Each alignment included the newly generated SSU rDNA sequences as well as sequences of closely related myxozoans available in GenBank, which were retrieved using BLASTN. Nucleotide sequences were aligned using MAFFT version 7.490 (^29^) implemented in Geneious Prime R11.1 (https://www.geneious.com), using the E-INS-i algorithm, with a gap opening penalty of 2.0. Alignments were edited to remove highly variable sections. Maximum likelihood (ML) analyses were performed using RAxML v7.2.8^30^ with the GTR + Γ model of nucleotide substitution with alpha parameter 0.5895 for the oligochaete-infecting lineage and 0.4924 for the polychaete-infecting lineage. The model was selected using jModelTest v2.1.10^31^. Bootstrap support values were calculated from 1000 replicas. Bayesian inference (BI) was performed using MrBayes v3.0^32^ with the GTR + Γ model of evolution and the same alpha parameters as for ML. MrBayes was run to estimate posterior probabilities over two million generations via two independent runs of four simultaneous Markov Chain Monte Carlo (MCMC) algorithms with every 100th tree saved. Tracer v1.4.1^33^ was used to set the length of the burn-in period and to identify potential convergence issues. Species-specific divergences were identified from proportional distances (in %) calculated in Geneious Prime based on the dataset used for the ML analysis.

## Results

### Prevalence and detection

**Figure 2:**
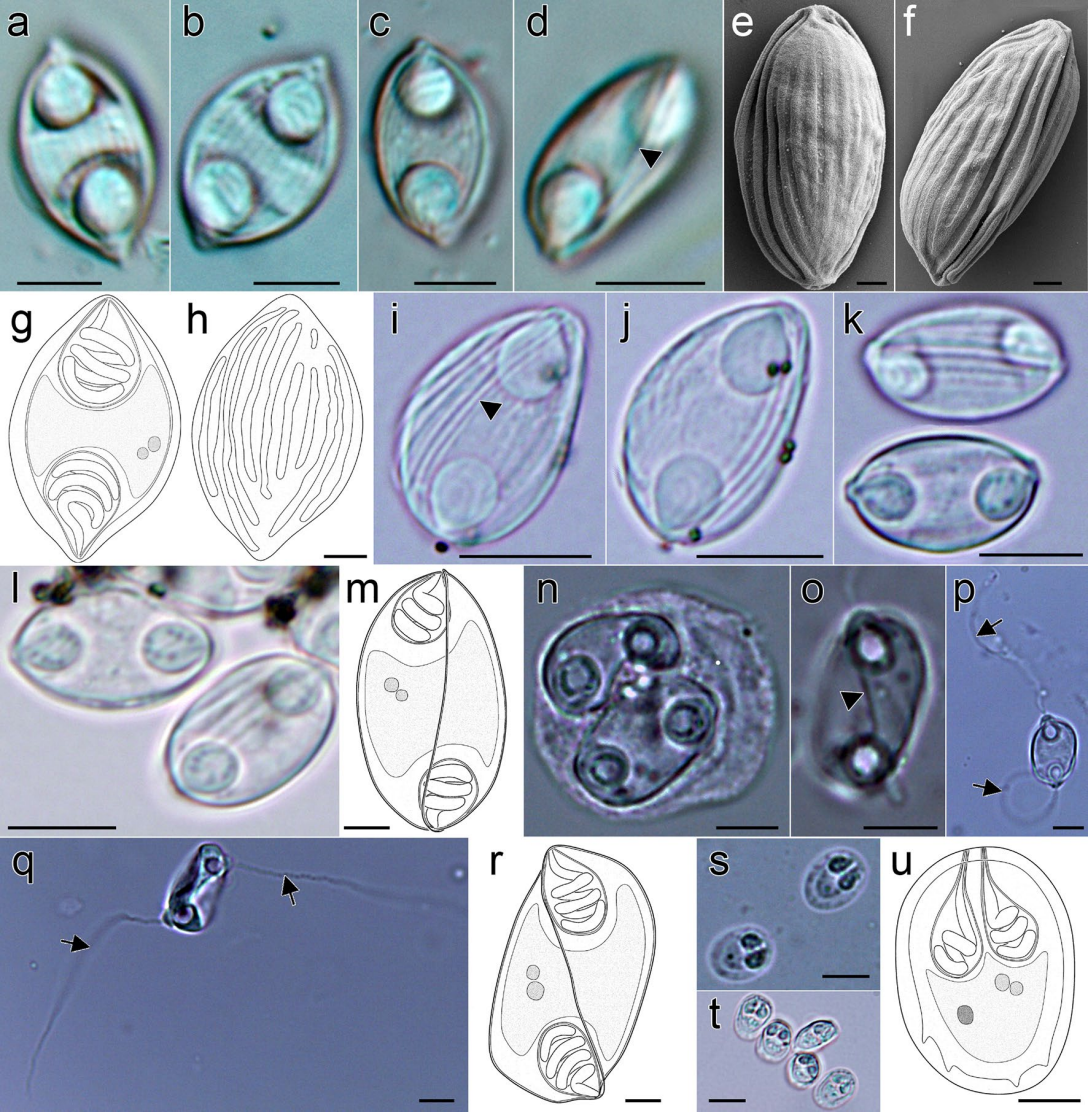
Myxospores morphology of the newly described myxozoan species of Neotropical fishes from Mexico. **(a–h)** Myxospores of *Myxidium zapotecus* sp. n. **(a–c)** Valvular view, showing myxospore width variability and surface ridges, **(d)** sutural view, **(e–f)** surface ridges pattern in valvular **(e)** and sutural **(f)** views and **(g–h)** schematic drawings of the myxospore, including ornamented valve, valvular views; **(i–m)** Myxospores of *Zschokkella guelaguetza* sp. n. **(i-j)** Surface ridges and suture, **(k)** sutural view, two different planes, **(l)** sutural (left) and valvular (right) views of myxospores and **(m)** schematic drawing of the myxospore, sutural view; **(n–r)** Plasmodia and myxospores of *Ellipsomyxa papantla* sp. n. **(n)** Disporic plasmodia, **(o)** myxospore, sutural view, **(p)** myxospore, valvular view, opposed extruded polar filaments, **(q)** myxospore, sutural view, opposed extruded polar filaments and **(r)** schematic drawing of the myxospore, sutural view; **(s–u)** Myxospores of *Myxobolus zoqueus* sp. n. **(s)** Two myxospores in valvular view, **(t)** five myxospores in several orientations and **(u)** schematic drawing of the myxospore, valvular view. a-d, i-l, t: 10% formalin fixed myxospores; n–q, s: fresh myxospores. a–d, i–l, n–q, s–t: light microscopy, scale bar: 5 µm; e–f: fixed in 2.5% glutaraldehyde in 0.1 M PBS, scanning electron microscopy, scale bar: 1 µm; g–h, m, r, u: drawings, scale bar: 2 µm. Suture—head arrow, extruded polar filament—arrow.

**Figure 3:**
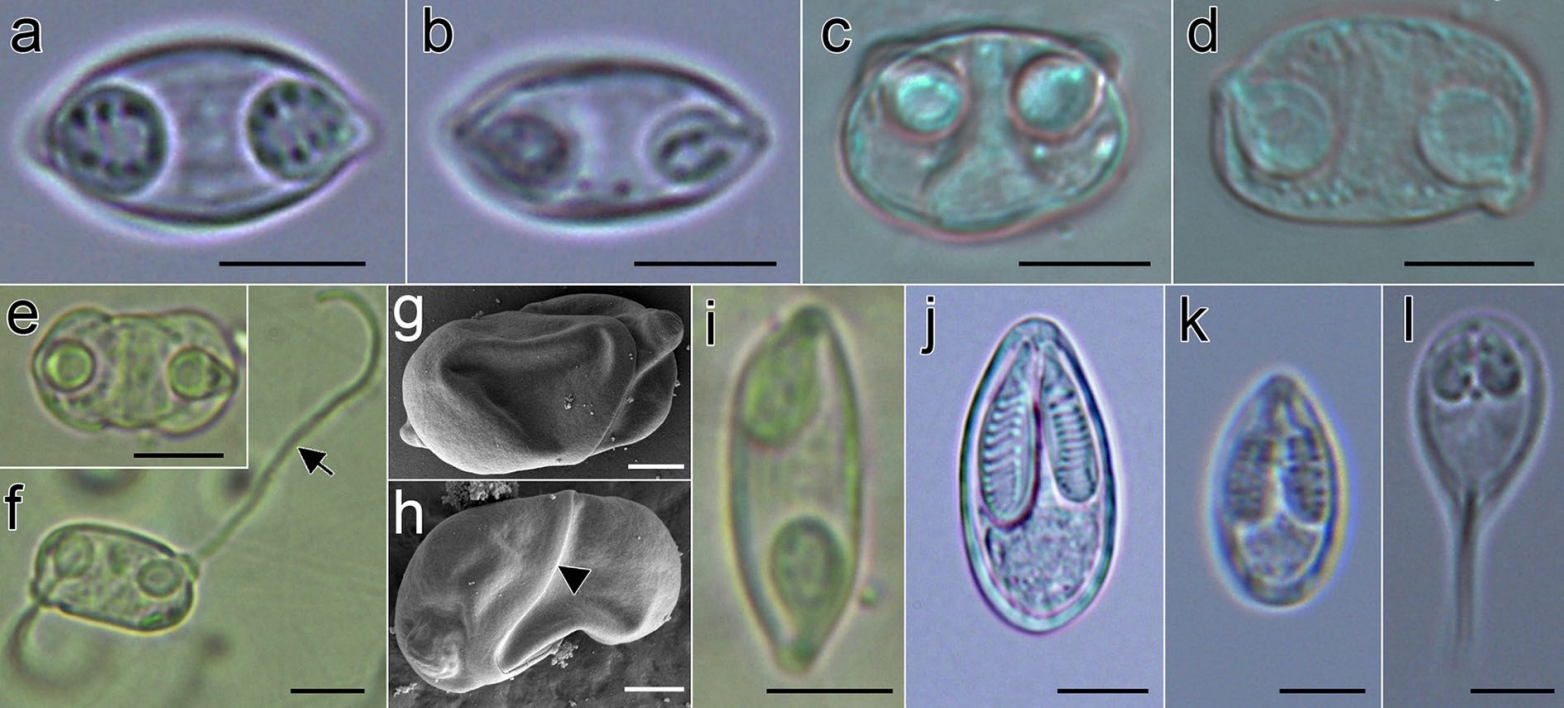
Myxospores morphology of undescribed species and mixed infections of Neotropical fishes from Mexico. **(a-b)** Mixed infection in *Awaous banana* (N19), **(a)** *Myxidium zapotecus* sp. n., **(b)** *Zschokkella* sp. ex *A. banana*; **(c-d)** Mixed infection in *Dormitator maculatus* gall bladder (P5), **(c)** *Zschokkella* sp. ex *D. maculatus*, **(d)** *Ellipsomyxa papantla* sp. n.; **(e–h)** *Ellipsomyxa* sp. ex *Dormitator maculatus* (L4)*,* **(e)** valvular view, **(f)** sutural view with opposed extruded polar filaments, **(g)** valvular view (partially collapsed valve), showing valvular protrusions associated to both polar capsules pointing out in opposite directions and **(h)** sutural view of smooth valves, with small valvular protrusion, where the opening and tip of the polar capsule its located; **(i)** *Myxidium* sp. ex *Paraneetroplus* sp. (C12); **(j)** *Myxobolus* sp. ex *Cichlasoma urophthalmus* (N1); **(k)** *Myxobolus* sp. ex *Profundulus punctatus* (N23); **(l)** *Henneguya* sp. ex *Parachromis friedrichsthalii* (N13). a–b, k–l: fresh myxospores; c–d, e–f, i–j: 10% formalin fixed myxospores; a–f, i–l: light microscopy, scale bar: 5 µm; g–h: fixed in 2.5% glutaraldehyde in 0.1M PBS, Scanning electron microscopy, scale bar: 2 µm. Suture—head arrow, extruded polar filament—arrow.

Microscopic examination showed that 16% (17/106) of the gall bladders were positive for myxosporean infection (Table 1). Myxozoan prevalence based on microscopic observations ranged from 14.3 to 100% among fish species. Eleven different species/morphotypes could be detected (Tables 1 and 2, Figs. 2 and 3), four of which are described below. Molecular screening revealed that 49% (50/102) of the gall bladder samples were positive for myxozoans, with prevalence of infection ranging from 25 to 100% among the analyzed fish species. None of the intestine and kidney samples were microscopically positive. Molecularly, 33.3% (3/9) of intestine and 11.1% (1/9) kidney samples were positive.

**Table 2:** Morphological and molecular data of mixed infections and undescribed species from gall bladder of fish hosts collected from Mexico. Species codes in comments column are according to Table 3. Abbreviations: L, length, W, width, T, thickness, PTL – polar tubule length, PTC polar tubule coils, FS – fresh spores; FFS – formalin fixed spores. All measurements are in µm.

Host (fish code)—locality	Genus or species (spore morphology)	Spore (L, W, T)	Polar capsule (L, W, PTL, PTC)	GenBank Acc. Number	Comments
*Awaous banana* (N19)—Río Grande, Matías Romero, Oaxaca	*Myxidium zapotecus* sp. n	13.1–13.3, 7.1–7.5 (n = 3); FS (Fig. 3a)	4.4–5.0, 3.8–4.1 (n = 6), 3 coils	OQ888233 & OQ888240	Mixed infection: *M. zapotecus* sp. n. + OBTC_species 7
	*Zschokkella* sp.	10.5–11.9 (n = 6), 6.0–6.2 (n = 2), 6.0–6.3 (n = 4); FS (Fig. 3b)	2.8–4.1, 2.6–3.3 (n = 12), 3 coils		
*Dormitator maculatus* (P5)—Tlacotalpan, Veracruz	*Zschokkella* sp.	– (Fig. 3c)	–	OQ888286 OQ888247 & OQ888232	Mixed infection: *E. papantla* sp.n. + OBTC_species_4
	*Ellipsomyxa papantla* sp. n	12.2 – 16.6 (n = 19), 8.3–10.6 (n = 6), 7.4–10.8 (n = 13); FFS (Fig. 3d)	3.3–5.8, 3.0–4.9 (n = 38), 19.4–54.9 (n = 4), 3–4 coils		
*Dormitator maculatus* (L4) – Tlacotalpan, Veracruz	*Ellipsomyxa* sp.	11.3–14.4 (n = 30), 7.3–8.9 (n = 14), 6.9–8.6 (n = 16); FFS (Fig. 3e-h)	3.1–4.5 (n = 56), 2.4–4.0 (n = 56), 6.3–29.5 (n = 13), 3–4 coils	OQ888222	Conflicting morphology and sequence data: OBTC_species_4
*Paraneetroplus* sp. (C12) -Río Negro, Santa María Chimalapa, Oaxaca	*Myxidium* sp.	14.4–15.8 (n = 12), 5.4–5.9 (n = 6), 5.4–6.2 (n = 6); FFS (Fig. 3i)	4.5–6.3, 2.8–3.7 (n = 24), 3 coils	OQ888273 & OQ888296 OQ888261 OQ888235	Mixed infection: OUTC_species_3 + OMsCVII_species_17 + OBTC_species_6
*Cichlasoma urophthalmus* (N1)—Tlacotalpan, Veracruz	*Myxobolus* sp.	– (Fig. 3j)	–	OQ888269 & OQ888252	Mixed infection: OMsCVII_species_8 + OMsCI_species_3
*Profundulus punctatus* (N23)—Santa María, Oaxaca	*Myxobolus* sp.	– (Fig. 3k)	–	OQ888267	Insufficient morphological data: OMsCVII_species_3
*Vieja fenestrata* (N11)—Río Grande, Matías Romero, Oaxaca	*Henneguya* sp.	Only field observations	–	OQ888268 OQ888243	Mixed infection & no morphological data OMsCVII_species_8 + OUTC_species_5
*Parachromis friedrichsthalii* (N13)—Río Grande, Matías Romero, Oaxaca	*Henneguya* sp.	– (Fig. 3l)	–	OQ888277	Conflicting morphology and sequence data: OUTC_species_2

The most frequently collected fish host species were three cyprinodontiforms, *Profundulus punctatus* (Profundulidae) (n = 23), *Profundulus oaxacae* (n = 14) and *Poecilia mexicana* (Poeciliidae) (n = 8); one mugiliform, *Dajaus monticola* (Mugilidae) (n = 7); and one perciform*, Dormitator maculatus* (Eleotridae) (n = 11). Myxozoan prevalence in the gall bladder of these hosts ranged from no detection to 27.3% microscopically, and from 25 to 85.7% by PCR detection (Table 1). Congruent prevalence of infection between microscopic examination and molecular screening was only observed in *Awaous banana* (Gobiidae) (100%) i.e., all three specimens analyzed showed myxospores in the bile. In some cases, microscopy did not reveal infection by myxospores, but PCR detected the presence of myxozoans up to 66% in some fish host species (*e.g.,* 66.7% in *Mayaheros urophthalmus* (Cichlidae), 50% in *Poecilia mexicana*, see Table 1). Overall, molecular screening increased myxozoan detection rates by 1.7 × to 6×. The only two myxozoan negative fish species were *Tlaloc labialis* (Profundulidae) (n = 1) and *Cichlasoma trimaculatum* (Cichlidae*)* (n = 1).

### Description of new species

Phylum Cnidaria Hatschek, 1888.

Unranked subphylum Myxozoa Grassé, 1970.

Class Myxosporea Bütschli, 1881.

Order Bivalvulida Schulman, 1959.

Suborder Variisporina Lom et Noble, 1984.

Family Myxidiidae Thélohan, 1892.

Genus *Myxidium* Bütschli, 1882.

### *Myxidium zapotecus* sp. n

#### Description of myxospores

Based on 18 myxospores fixed in formalin from the bile of one host (code: C8). Data obtained using light microscopy and SEM. Myxospores fusiform in valvular view, ellipsoidal in sutural view (Fig. 2a–h) measuring 12.5 ± 1.3 (10.8**–**14.6; n = 18) in length, 8.6 ± 1.2 (6.9**–**11.3; n = 10) in width and 6.3 ± 0.5 (5.6**–**7.1; n = 8) in thickness, with pointed ends. Two valves with 9**–**10 longitudinal surface ridges (Fig. 2e, f and h), parallel to sutural plane. Suture bisects the myxospore. Two equal subspherical polar capsules, 4.2 ± 0.6 (3.1**–**5.4; n = 35) long and 3.4 ± 0.6 (2.6**–**4.6; n = 35) wide (Fig. 2a–d, g). Polar capsules positioned at opposite ends of the myxospore and opening to different sides in sutural plane. Polar tubule arranged in 3 coils. Sporoplasm binucleate, in middle of myxospore.

### Taxonomic summary

*Type host*: River goby *Awaous banana* (Valenciennes) (Gobiiformes: Gobiidae).

*Type locality*: Río Negro, Santa María Chimalapa (16°53'55''N; 94°41'37'' W), Oaxaca, Mexico.

*Other locality*: Río Grande, Matías Romero (16°47'29"N; 95°01'02"W), Oaxaca, Mexico.

*Additional hosts and localities*: Mountain mullet *Dajaus monticola* (Bancroft) (Mugiliformes: Mugilidae) ex Río Grande, Matías Romero (16°47'29"N; 95°01'02" W), Oaxaca, Mexico; *Astyanax* sp. (Characiformes: Characidae) ex Río Negro, Santa María Chimalapa (16°53'55'' N; 94°41'37'' W), Oaxaca, Mexico.

*Site in hosts*: Gall bladder.

*Prevalence in type host:* 66.6 % (2/3) microscopic detection (1/1—Type locality, 1/2—Other locality), 66.6% (2/3) molecular detection.

*Prevalence in other hosts:* 33.3% (1/3) in *Dajaus monticola* and 33.3% (1/3) in *Astyanax* sp. (molecular detection only).

*Material deposited:* Kwik-Diff stained slides of air-dried myxospores (C8) (CNHE 11950).

*Representative SSU rDNA sequences:* GenBank OQ888226 ex *Awaous banana* (1,806 bp; code: C8); OQ888240 ex *A. banana* (919 bp; code HP179_N19); OQ888239 ex *Dajaus monticola* (920 bp; code: HP183_N24); OQ888242 and OQ888241 ex *Astyanax* sp. (919 and 919 bp; code: HP121_C10 & HP123_C10).

ZooBank* registration*: The Life Science Identifier (LSID) of the article is urn:lsid:zoobank.org:pub:D74C1323-E34D-4D7A-816E-098EA48188C4. The LSID for the new name *Myxidium zapotecus* sp. n. is urn:lsid:zoobank.org:act: 46A34393-6842-4319-87EF-593D17A69837.

*Etymology:* The species is named after the indigenous pre-Columbian civilization in the Valley of Oaxaca in Mesoamerica, the Zapotecs.

### Remarks

Myxospore measurements of *Myxidium zapotecus* sp. n. from another specimen of *Awaous banana* (N19) are provided in Table 2 (Fig. 3a). In this host, a mixed infection was detected with a *Zschokkella* sp. (Fig. 3b). *Myxidium zapotecus* sp. n. has overall myxospore morphology characteristic of the genus *Myxidium*. For differential diagnosis we selected species of *Myxidium* reported from North America, from other gobiids and other species that showed sequence similarity to our new species^34^ (Supplementary Data 4). The new species can be distinguished from its congeners based on the fish intermediate host and/or geographic location. *Myxidium zapotecus* sp. n. differs in myxospore shape to all the species compared, except for *Myxidium phyllium* (Davis, 1917), *Myxidium truttae* Léger, 1930 and *Myxidium eminentis* Ishizaki, 1957. These four species shared a fusiform shape in valvular view, in contrast to the other species with oval, rounded, more ellipsoid myxospores. The new species differs in both myxospore length and width to *Myxidium coryphaenoidium* Noble, 1966, *Myxidium amazonense* Mathews, Silva, Maia & Adriano, 2015 and *Myxidium whippsi* McAllister, Cloutman, Leis & Robison, 2022, and in myxospore width to *Myxidium pseudocuneiforme* Chen, Zhang, Whipps, Yang & Zhao, 2021 and *Myxidium kudoi* Meglitsch, 1937. It differs in polar capsule shape to *Myxidium glossogobi* Chakravarty, 1939 and *Myxidium pseudocuneiforme* (subspherical vs pyriform). The new species differs in number of polar tubule coils to *Myxidium coryphaenoidium, Myxidium amazonense* and *Myxidium pseudocuneiforme* (3 coils vs. > 4 coils; Supplementary Data 4) and in number of valve striations to *Myxidium pseudocuneiforme* (9–10 vs. 6–8), *Myxidium whippsi* (9–10 vs. 5) and *Myxidium glossogobi* (9–10 vs. no striations).

The new species overlaps in myxospore size with *Myxidium phyllium*, *Myxidium truttae* and *Myxidium eminentis*. However, *Myxidium zapotecus* sp. n. has longer and wider myxospores and longer polar capsules than *Myxidium phyllium* and *Myxidium truttae* and differs in fish host (river goby *Awaous banana* vs mosquitofish *Gambusia affinis* and brown trout *Salmo trutta*, respectively) and in geographic location (Mexico vs USA and France, respectively). *Myxidium zapotecus* sp. n. differs from *Myxidium eminentis* in myxospore width, fish host (river goby *Awaous banana* vs flat-headed goby *Luciogobius guttatus*) and geographic location (Mexico vs Japan).

Genus *Zschokkella* Auerbach, 1910.

### *Zschokkella guelaguetza* sp. n

#### Description of myxospores

Based on 25 formalin fixed myxospores from one host (code: N26) by light microscopy. Myxospores ellipsoidal in sutural view and slightly semicircular in valvular view (Fig. 2i–m) measuring 10.9 ± 0.4 (9.1**–**11.9; n = 25) in length, 6.2 ± 0.6 (5.6**–**7.5: n = 16) in width, 7.0 ± 0.4 (6.1**–**7.3, n = 9) thickness, with blunted ends. Two unequal valves, with longitudinal surface ridges, sinuous suture (Fig. 2i–k). Two equal subspherical polar capsules, 3.2 ± 0.3 (2.6**–**3.7: n = 50) long and 2.8 ± 0.2 (2.2**–**3.2: n = 50) wide. Polar capsules subterminal to one side/valve (Fig. 2m). Polar tubule arranged in 2**–**3 coils. Sporoplasm binucleate, in middle of myxospore.

### Taxonomic summary

*Type host:* River goby *Awaous banana* (Valenciennes) (Gobiiformes: Gobiidae).

*Type locality:* Río Grande, Matías Romero (16°47'29"N; 95°01'02"W), Oaxaca, Mexico.

*Other host and locality: Astyanax* sp. (Characiformes: Characidae) ex Río Negro, Santa María Chimalapa (16°53'55''N; 94°41'37''W), Oaxaca, Mexico.

*Site in hosts:* Gall bladder.

*Prevalence in type host*: 33.3% (1/3) microscopic detection, 33.3% (1/3) molecular detection.

*Prevalence in other host:* 33.3% (1/3) in *Astyanax* sp. (molecular detection only).

*Material deposited:* Kwik-Diff stained slides of air-dried myxospores (N26) (CNHE 11951).

*Representative SSU rDNA sequences:* GenBank OQ888223 ex *Awaous banana* (1,926 bp; code: N26); OQ888237 ex *Astyanax* sp. (993 bp; code: HP122_C10).

*ZooBank registration*: The Life Science Identifier (LSID) of the article is urn:lsid:zoobank.org:pub: D74C1323-E34D-4D7A-816E-098EA48188C4. The LSID for the new name *Zschokkella guelaguetza* sp. n. is urn:lsid:zoobank.org:act: 2BC5693F-D700-4B00-8D76-46E0C467B55E.

*Etymology:* The species is named after the colorful festivity of La Guelaguetza, an annual indigenous cultural dancing event that takes place in Oaxaca, Mexico.

### Remarks

The novel species has overall myxospore morphology characteristics of the genus *Zschokkella*. For differential diagnosis we selected species of *Zschokkella* reported in gobiids and other fish species that showed sequence similarity to the new species^35^. *Zschokkella guelaguetza* sp. n. can be distinguished from other *Zschokkella* species by fish alternate host and geographical location (Supplementary Data 4). The new species differs in myxospore shape to *Zschokkella fujitai* Ozaki & Isizaki, 1941, *Zschokkella glossogobii* Kalavati & Vaidehi, 1991, *Zschokkella nova* Klokačewa, 1914 and *Zschokkella trachini* Azizi, Rangel, Castro, Santos & Bahri, 2016 (Ellipsoidal vs Ovoid, oval or elongate myxospore in sutural view). It differs in myxospore length and width to *Zschokkella trachini* and *Zschokkella soleae* Yemmen, Marton, Bahri & Eszterbauer, 2013, and in myxospore length to *Zschokkella fujitai*. The new species differs in polar capsule shape to all species (i.e. subspherical vs pyriform, ovoid or spherical), except to *Zschokkella auratis* Rocha, Casal, Rangel, Severino, Castro, Azevedo & Santos, 2013, *Zschokkella fujitai* and *Zschokkella balistoidi* Heiniger & Adlard, 2014. *Zschokkella guelaguetza* sp. n. differs in polar capsule length and width to *Zschokkella fujitai* and to *Zschokkella glossogobii* and in number of polar tubule coils to *Zschokkella soleae* and *Zschokkella auratis* (2 to 3 coils vs 4 to 5 coils).

*Zschokkella guelaguetza* sp. n. overlaps in myxospore size with *Zschokkella balistoidi* and *Zschokkella gobidiensis* Sarkar & Ghosh, 1991. The new species has slightly longer and wider polar capsules than *Zschokkella balistoidi* and differs in host (river goby *Awaous banana* vs titan triggerfish *Balistoides viridescens*) and geographic location (Mexico vs Australia). *Zschokkella guelaguetza* sp. n. can be differentiated from *Zschokkella gobidiensis* on having shorter and narrower myxospores, on fish intermediate host (river goby *Awaous banana* vs tank goby *Glossogobius giuris*) and on geographic location (Mexico vs India).

Family Ceratomyxidae Doflein, 1899.

Genus *Ellipsomyxa* (Køie, 2003).

### *Ellipsomyxa papantla* sp. n

#### Description of myxospores

Based on 19 fresh myxospores from one host (code: N4) by light microscopy. Myxospores ovoid in valvular view and ellipsoidal in sutural view (Fig. 2n–r) measuring 12.9 ± 0.8 (11.6**–**15.0; n = 19) in length, 9.1 ± 0.5 (7.6**–**9.9; n = 7) in width and 7.3 ± 0.7 (6.1**–**8.2; n = 12) in thickness. Two smooth valves, transverse suture, forming acute angle to thickness (Fig. 2o). Small valvular protrusions associated with tips of polar capsules (Fig. 2p,q). Two equal spherical to pyriform polar capsules, 3.8 ± 0.5 (2.6**–**4.6; n = 25) long and 3.3 ± 0.5 (2.2**–**4.2: n = 25) wide. Polar capsules close to sutural plane, discharging at opposite sides (Fig. 2p,q). Polar tubule measuring 20.9**–**29.2 in length (n = 6), arranged in 3**–**4 coils. Sporoplasm binucleate, in middle of myxospore. Disporic plasmodia (Fig. 2n).

### Taxonomic summary

*Type host:* Fat sleeper *Dormitator maculatus* (Bloch) (Gobiiformes: Eleotridae).

*Type locality:* Tlacotalpan (18º36'41''N; 95º39'44''W), Veracruz, Mexico.

*Site in host:* Gall bladder.

*Prevalence:* 18.2% (2/11) microscopic detection, 30% (3/10) molecular detection.

*Material deposited:* Kwik-Diff stained slides of air-dried myxospores (N4) (CNHE 11952).

*Representative SSU rDNA sequences:* GenBank OQ888230 ex *Dormitator maculatus* (1,603 bp; code: N4); OQ888285 ex *D. maculatus* (761 bp; code: HP92_J5), and OQ888286 ex *D. maculatus* (761 bp; code: HP89_P5).

*ZooBank registration*: The Life Science Identifier (LSID) of the article is urn:lsid:zoobank.org:pub: D74C1323-E34D-4D7A-816E-098EA48188C4. The LSID for the new name *Ellipsomyxa papantla* sp. n. is urn:lsid:zoobank.org:act: F901367A-FCBE-44F1-8392-026367CEA598.

*Etymology:* The species is named after one of the localities where the ancient Mesoamerican ritual ceremony, “Danza de los Voladores” takes place - Papantla, Veracruz, Mexico.

### Remarks

Measurements for formalin-fixed *Ellipsomyxa papantla* sp. n. myxospores detected in another specimen of *D. maculatus* (P5) are provided in Table 2 (Fig. 3d). In this host, a mixed infection with a *Zschokkella* sp. was observed (see Fig. 3c). *Ellipsomyxa papantla* sp. n. has overall myxospore morphology characteristic of the genus *Ellipsomyxa*. For differential diagnosis we selected all species of *Ellipsomyxa* reported to date (Supplementary Data 4). The novel species is unique with respect to all other *Ellipsomyxa* species in fish host and geographic location. It differs in myxospore shape to all the species compared, except to *Ellipsomyxa apogoni* Heiniger & Adlard, 2014; both species having ellipsoidal myxospores in sutural view and ovoid in valvular view. *Ellipsomyxa papantla* sp. n. myxospores differ in length and width to *Ellipsomyxa gobioides* Azevedo, Videira, Casal, Matos, Oliveira, Al-Quraishy & Matos, 2013 and *Ellipsomyxa boleophthalmi* Vandana, Poojary, Tripathi, Pavan-Kumar, Pratapa, Sanil & Rajendran, 2021. It differs in myxospore length to *Ellipsomyxa fusiformis* (Davis, 1917), *Ellipsomyxa gobii* Køie, 2003, *Ellipsomyxa syngnathi* Køie & Karlsbakk, 2009, *Ellipsomyxa apogoni* Heiniger & Adlard, 2014 and *Ellipsomyxa tucujuensis* Ferreira, da Silva, de Carvalho, Bittencourt, Hamoy, Matos, & Videira, 2021. *Ellipsomyxa papantla* sp. n. differs in myxospore width to *Ellipsomyxa arariensis* Da Silva, Matos, Lima, Furtado, Hamoy & Matos, 2018, *Ellipsomyxa arothroni* Heiniger & Adlard, 2014, *Ellipsomyxa kalthoumi* Thabet, Tlig-Zouari, Al Omar & Mansour, 2016, *Ellipsomyxa manilensis* Heiniger & Adlard, 2014 and *Ellipsomyxa plagioscioni* Zatti, Maia & Adriano, 2020. The new species differs in polar capsule length to *Ellipsomyxa arothroni* and *Ellipsomyxa kalthoumi*, and in polar capsule length and width to *Ellipsomyxa manilensis*. It differs in the number of polar tubule coils to *Ellipsomyxa adlardi* Whipps & Font, 2013, *Ellipsomyxa arariensis*, *Ellipsomyxa gobii*, *Ellipsomyxa gobioides*, *Ellipsomyxa kalthoumi*, *Ellipsomyxa plagioscioni*, *Ellipsomyxa mugilis* (Sitja-Bobadilla & Alvarez-Pellitero, 1993), *Ellipsomyxa nigropunctatis* Heiniger & Adlard, 2014, *Ellipsomyxa syngnathi* and *Ellipsomyxa tucujuensis* (3 to 4 vs. > 5 coils).

The new species overlaps in myxospore size with *Ellipsomyxa amazonensis* Zatti, Atkinson, Maia, Correa, Bartholomew & Adriano, 2018, *Ellipsomyxa ariusi* Chandran, Zacharia, Sathianandan & Sanil, 2020 and *Ellipsomyxa paraensis* Zatti, Maia & Adriano, 2020. However, *Ellipsomyxa papantla* sp. n. has longer and wider myxospores than these three species, as well as wider polar capsules, and differs in host range (Fat sleeper *Dormitator maculatus* vs gilded catfish *Brachyplatystoma rousseauxii*, threadfin sea catfish *Arius arius* and tucunaré *Cichla monoculus*) and geographic location (Mexico vs Brazil and India).

Suborder Platysporina Kudo, 1919.

Family Myxobolidae Thélohan, 1892.

Genus *Myxobolus* Bütschli, 1881.

### *Myxobolus zoqueus* sp. n

#### Description of myxospores

Based on 30 fresh myxospores from one host (code: N21) by light microscopy. Myxospores oval in valvular view, ellipsoidal in sutural view (Fig. 2s–u) measuring 7.9 ± 0.3 (7.1**–**8.6; n = 30) in length, 5.9 ± 0.4 (4.7**–**6.5; n = 30) in width. Two smooth valves, suture straight. Four to five notches at sutural edge occasionally evident, at posterior part (Fig. 2s,u). Two equal pyriform polar capsules 2.6 ± 0.3 (2.0**–**3.1: n = 58) long and 1.6 ± 0.2 (1.1**–**2.2; n = 58) wide, opening at anterior part. Polar tubule arranged in 2**–**3 coils. Binucleate sporoplasm at posterior part of the myxospore with iodinophilous vacuole.

### Taxonomic summary

*Type host:* Mountain mullet *Dajaus monticola* (Bancroft) (Mugiliformes: Mugilidae).

*Type locality:* Río Grande, Matías Romero (16°47'29" N; 95°01'02" W), Oaxaca, Mexico.

*Site in host:* Gall bladder.

*Prevalence:* 33.3% (1/3) microscopic detection, 66.6% (2/3) molecular detection.

*Material deposited:* Kwik-Diff stained slides of air-dried myxospores (N21) (CNHE 11953).

*Representative SSU rDNA sequences:* GenBank OQ888227 ex *Dajaus monticola* (1,804 bp; code: N21); and OQ888253 ex *D. monticola* (863 bp; code: HP182_N24).

*ZooBank registration*: The Life Science Identifier (LSID) of the article is urn:lsid:zoobank.org:pub: D74C1323-E34D-4D7A-816E-098EA48188C4. The LSID for the new name *Myxobolus zoqueus* sp. n. is urn:lsid:zoobank.org:act: A99526D1-9F6D-421F-A4D9-52C0DEAA60C3.

Etymology: The species is named after the Zoque people, an indigenous ethnic group present in Oaxaca, Chiapas and Tabasco in Mexico.

### Remarks

*Myxobolus zoqueus* sp. n. has overall myxospore morphology characteristics of the genus *Myxobolus*. For differential diagnosis we selected species of *Myxobolus* reported from Mexico and from other fish intermediate hosts belonging to the order Mugiliformes (Supplementary Data 4). *Myxobolus zoqueus* sp. n. can be distinguished from its congeners based on fish host species and site of infection (gall bladder). The new species differs in myxospore length to *Myxobolus curema* Vieira, Agostinho, Negrelli, da Silva, de Azevedo & Abdallah, 2022, *Myxobolus pupkoi* Gupta, Haddas-Sasson, Gayer & Huchon, 2022 and *Myxobolus nuevoleonensis* Segovia-Salinas, Jiménez-Guzmán, Galaviz-Silva & Ramírez-Bon, 1991. It differs in myxospore width to *Myxobolus exiguus* (Thélohan, 1895), *Myxobolus peritonaeum* Rocha, Casal, Alves, Antunes, Rodrigues & Azevedo, 2019 and *Myxobolus ramadus* Rocha, Casal, Alves, Antunes, Rodrigues & Azevedo, 2019. *Myxobolus zoqueus* sp. n. differs in both polar capsule length and width to *Myxobolus cartilaginis* Hoffman, Putz & Dunbar, 1965, *Myxobolus exiguus*, *Myxobolus nuevoleonensis* and *Myxobolus ramadus*, and differs in polar capsule length to *Myxobolus muscularis* Rocha, Casal, Alves, Antunes, Rodrigues & Azevedo, 2019 and *Myxobolus peritonaeum* Rocha, Casal, Alves, Antunes, Rodrigues & Azevedo, 2019. The new species differs in the number of coils of the polar tubule (2 to 3 vs. > 4 and up to 11) and in the number of sutural folds (4 to 5 vs. > 6 up to 10) to all compared species.

*Myxobolus zoqueus* sp. n. overlaps in myxospore size to *Myxobolus mexicanus* Yoshino & Noble, 1973. However, the new species has shorter and narrower myxospores, as well as shorter polar capsules. *Myxobolus zoqueus* sp. n. also differs in polar capsule positions, which was described as ‘anteriorly located and shifted to one side of the myxospore's valvular midline’ for *Myxobolus mexicanus*^36^. In addition, the new species differ from *Myxobolus mexicanus* by its fish host (mountain mullet *Dajaus monticola* vs shoulderspot grenadier *Coelorinchus scaphopsis*), site of infection (gall bladder vs kidney), geographic location (Oaxaca, southwestern Mexico vs Baja California, northwestern Mexico) and habitat (freshwater vs marine).

*Myxobolus zoqueus* sp. n. overlaps in myxospore size to the measurements provided for the species *Myxobolus cartilaginis* ex *Micropterus salmoides* from Mexico^10^. The new species is shorter and narrower than *Myxobolus cartilaginis* ex *Micropterus salmoides* and the polar capsule length differs between both species. Both species can also be distinguished by its fish host (mountain mullet *Dajaus monticola* vs largemouth black bass *Micropterus salmoides*), site of infection (gall bladder vs branchiostegal rays) and geographic location in Mexico (Oaxaca, southwestern Mexico vs Nuevo León, northeastern Mexico).

### Molecular and phylogenetic results

**Figure 4:**
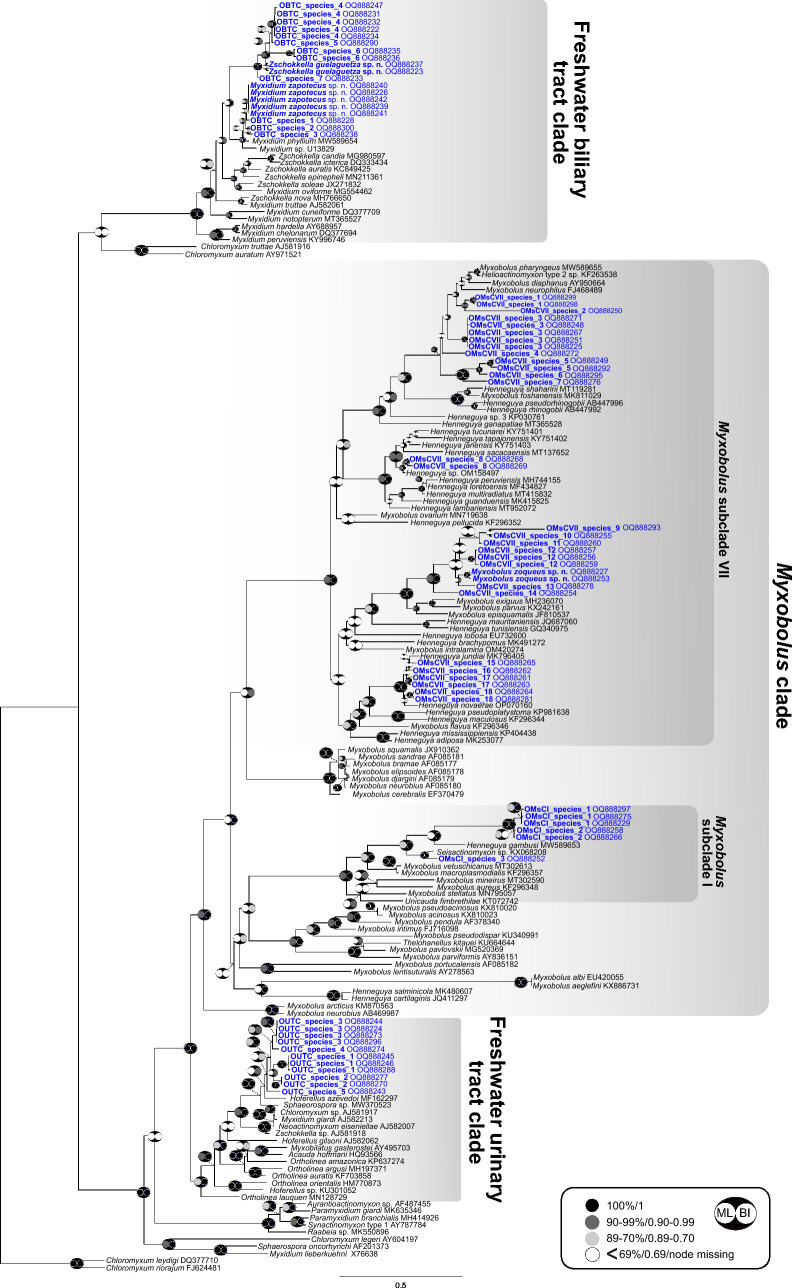
Phylogenetic position of newly identified SSU rDNA myxozoan sequences of Neotropical fishes from Mexico within the oligochaete-infecting lineage. The tree was inferred using maximum likelihood analyses. Bootstrap support and Bayesian posterior probabilities are indicated by pictograms at nodes (see legend). GenBank accession numbers are provided beside taxon names. Newly sequenced taxa are indicated in blue. A scale bar is included to visualize the branch lengths and evolutionary distances between different taxa.

**Figure 5:**
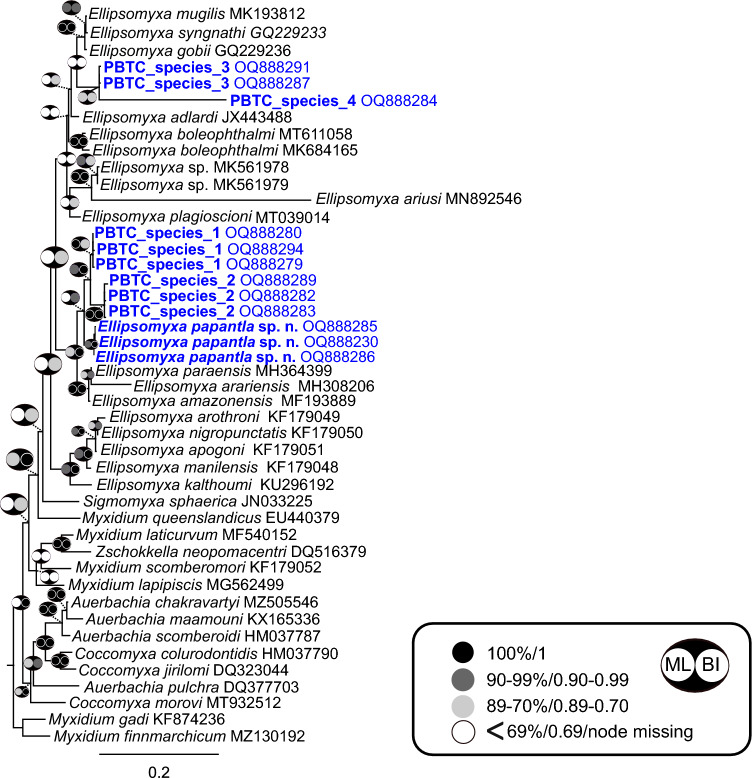
Phylogenetic position of newly identified SSU rDNA myxozoan sequences of Neotropical fishes from Mexico within the polychaete-infecting lineage. The tree was inferred using the maximum likelihood analyses. Bootstrap support and Bayesian posterior probabilities are indicated by pictograms at nodes (see legend). GenBank accession numbers are provided beside taxon names. Newly sequenced taxa are indicated in blue. A scale bar is included to visualize the branch lengths and evolutionary distances between different taxa.

A total of 79 novel partial SSU rDNA sequences were generated in this study (Supplementary Data 3). Sixty-seven sequences were representatives of the oligochaete-infecting lineage (mostly freshwater fish infecting species), belonging to three clades: urinary tract clade (11 sequences), biliary tract clade (19 sequences) and *Myxobolus* clade (37 sequences). Sequences falling in the *Myxobolus* clade were found to group in subclade I (6 sequences) and VII (31 sequences) (Fig. 4) (subclades according to^37^). Twelve sequences belonged to the polychaete-infecting lineage (mostly marine fish infecting species), specifically to the biliary tract clade and most likely represented species of *Ellipsomyxa* Køie, 2003 (Fig. 5). None of the newly generated sequences were conspecific to any species or sequence data available in GenBank.

Using the phylogenetic position (topology, branch length) and a threshold of > 2% genetic distances of the 79 novel sequences and their closest relatives, a total of 41 putative new species were identified (37 putative + 4 new species described above): five species in the oligochaete-infecting urinary tract clade, nine species in the oligochaete-infecting biliary tract clade, including *Myxidium zapotecus* sp. n. and *Zschokkella guelaguetza* sp. n. and 22 species in the *Myxobolus* clade, with three species belonging to the subclade I and 19 species to the subclade VII, including *Myxobolus zoqueus* sp. n. In the polychaete-infecting biliary tract clade, we identified five species, including the newly described *Ellipsomyxa papantla* sp. n. (Figs. 4, 5; Table 3).

**Table 3:** Novel and putative myxozoan species of Neotropical fishes from Mexico according to their SSU rDNA molecular data, phylogenetic position (topology, branch length) and genetic distances (> 2%). All species were found in gall bladder, except for four species detected in the intestine (*) and/or kidney ( +). Abbreviations: L – Lineage; CL – Clade; Species code: OUTC—Oligochaete-infecting urinary tract clade, OBTC – Oligochaete-infecting biliary tract clade, OMsC – Oligochaete-infecting *Myxobolus* clade, PBTC – Polychaete-infecting biliary tract clade. State: V – Veracruz, O – Oaxaca, Ch – Chiapas. % ID – percentage of identity. #BP – number of different base pairs.

L	CL	#	SPECIES CODE	HOSTS	State	GENBANK acc numbers	% id	#bp
Oligochaete-infecting	Urinary tract	1	OUTC_species_1	*Dormitator maculatus* *Sarda sarda*	V	OQ888246, OQ888245, OQ888288	99.5–99.6	3–4
		2	OUTC_species_2	*Parachromis friedrichsthalii* *Vieja zonata*	O	OQ888277, OQ888270	99.7	2
		3	OUTC_species_3	*Paraneetroplus bulleri* *Paraneetroplus* sp.	O	OQ888273, OQ888296, OQ888244, OQ888224	99.8	1
		4	OUTC_species_4	*Thorichthys maculipinnis*	O	OQ888274	–	–
		5	OUTC_species_5	*Vieja fenestrata*	O	OQ888243	–	–
	Biliary tract	6	*Myxidium zapotecus* sp. n.	*Awaous banana* *Dajaus monticola* *Astyanax* sp.	O	OQ888242, OQ888241, OQ888226, OQ888240, OQ888239	99.8–99.9	1–2
		7	OBTC_species_1	*Eleotris* sp.	V	OQ888228	–	–
		8	OBTC_species_2	*Synbranchus marmoratus*	O	OQ888300	–	–
		9	OBTC_species_3	*Synbranchus marmoratus*	O	OQ888238	–	–
		10	OBTC_species_4	*Dormitator maculatus*	V	OQ888231, OQ888222, OQ888234, OQ888247, OQ888232	99.3–100	0–6
		11	OBTC_species_5	*Gobiomorus dormitor*	V	OQ888290	–	–
		12	OBTC_species_6	*Paraneetroplus* sp.	O	OQ888235, OQ888236	99.2	8
		13	*Zschokkella guelaguetza* sp. n.	*Awaous banana* *Astyanax* sp.	O	OQ888237, OQ888223	98.8	12
		14	OBTC_species_7	*Awaous banana*	O	OQ888233	–	–
	*Myxobolus*	15	OMsCI_species_1	*Profundulus punctatus*	Ch	OQ888297, OQ888275, OQ888229	97.1–99.5	3–24
		16	OMsCI_species_2	*Profundulus oaxacae*	O	OQ888266, OQ888258	99.4	5
		17	OMsCI_species_3	*Mayaheros urophthalmus*	V	OQ888252	–	–
		18	OMsCVII_species_1	*Poecilia mexicana*	V	OQ888299, OQ888298	99.7	1
		19	OMsCVII_species_2	*Poecilia mexicana*	V	OQ888250	–	–
		20	OMsCVII_species_3	*Profundulus punctatus*	O, Ch	OQ888251, OQ888225, OQ888267, OQ888271, OQ888248	99.8–99.8	1–4
		21	OMsCVII_species_4	*Profundulus oaxacae*	O	OQ888272	–	–
		22	OMsCVII_species_5	*Poecilia mexicana* *Dormitator maculatus*(*)	V	OQ888249, OQ888292	97.9	15
		23	OMsCVII_species_6	*Xiphophorus* sp.	V	OQ888295	–	–
		24	OMsCVII_species_7	*Poecilia sphenops* (*)	V	OQ888276	–	–
		25	OMsCVII_species_8	*Mayaheros urophthalmus* *Vieja fenestrata*	V O	OQ888268, OQ888269	98.7	11
		26	OMsCVII_species_9	*Dajaus monticola*	V	OQ888293	–	–
		27	OMsCVII_species_10	*Dajaus monticola*	V	OQ888255	–	–
		28	OMsCVII_species_11	*Dajaus monticola*	V	OQ888260	–	–
		29	OMsCVII_species_12	*Xiphophorus alvarezi*	V	OQ888257, OQ888256, OQ888259	98.4	3–14
		30	*Myxobolus zoqueus* sp. n.	*Dajaus monticola*	O	OQ888227, OQ888253	99.6	3
		31	OMsCVII_species_13	*Dajaus monticola*	V	OQ888278	–	–
		32	OMsCVII_species_14	*Profundulus punctatus*	O	OQ888254	–	–
		33	OMsCVII_species_15	*Rhamdia quelen*	O	OQ888265	–	–
		34	OMsCVII_species_16	*Rhamdia quelen*	Ch	OQ888262	–	–
		35	OMsCVII_species_17	*Profundulus punctatus* *Paraneetroplus* sp.	Ch O	OQ888261, OQ888263	98.3	15
		36	OMsCVII_species_18	*Rhamdia guatemalensis* (*)( +)	V	OQ888281, OQ888264	99.6	5
Polychaete-infecting	Biliary tract	37	*Ellipsomyxa papantla* sp. n.	*Dormitator maculatus*	V	OQ888285, OQ888230, OQ888286	99.6–99.7	2–3
		38	PBTC_species_1	*Mayaheros urophthalmus* *Sarda sarda*	V	OQ888279, OQ888294, OQ888280	99.7–99.9	1–2
		39	PBTC_species_2	*Maskaheros regani* *Poecilia mexicana* *Xiphophorus alvarezi*	O V V	OQ888282, OQ888283, OQ888289	99.5–99.7	2–4
		40	PBTC_species_3	*Dajaus monticola*	V	OQ888287, OQ888291	99.7	2
		41	PBTC_species_4	*Dajaus monticola*	V	OQ888284	–	–

All five newly identified species from the oligochaete-infecting urinary tract clade clustered together with the closest relative *Hoferellus azevedoi* Matos, Silva, Hamoy & Matos, 2018 (MF162297) from the *Chaetobranchus azevedoi* from Marajo Island in northern Brazil. All nine species in the oligochaete-infecting biliary tract clade are closely related species forming two distinct phylogenetic groups with only one sequence of a described species available in GenBank, *Myxidium phyllium* (MW589654), which was found in gall bladder of *Gambussia affinis*, a cyprinodontiformid fish with a wide distribution range from North America to Central America. Two species clustering in the *Myxobolus* subclade I (OMsCI_species_1 & 2) are sister related to *Henneguya gambusi* Parker, Spall & Warner, 1917 (MW589653) from the aforementioned host *G. affinis.* The third species of *Myxobolus* subclade I (OMsCI_species_3) revealed the highest similarity to the sequence from actinosporean stage Seisactinomyxon (KX068208) from naidid annelid *Slavina evelinae* from Brazil. The rest of 19 putative myxozoan species are spread in four distinct phylogenetic groups within *Myxobolus* subclade VII suggesting wide diversity of phylogenetic lineages of Mexican myxozoans within this *Myxobolus* subgroup. Four putative species (OMsCVII_species_15-18) clustered within the group of myxobolids infecting silurid fish in the subclade VII. The closest relative appeared to be *Henneguya jundiai* Negrelli, Vieira, Tagliavini, Abdallah & de Azevedo, 2019 (MK796405) and *Henneguya novaerae* Mirandola Dias Vieira, Bravin Narciso & da Silva, 2022 (OP070160) from silurid *Rhamdia quelen* from Brazil, the same fish host as for two new putative species from Mexico (OMsCVII_species_15 & 16). The second group of six closely related putative species and *Myxobolus zoqueus* sp. n. clustering with *Myxobolus* subgroup VII was phylogenetically placed in the mugilid infecting myxobolids. This host preference agrees with our finding that five of the newly identified species are from the mugilid *Dajaus monticola*. The putative species OMsCVII_species_8 isolated from two cichlid fish clustered in a close relation with *Henneguya* sp. (OM158497) infecting cichlids from Amazon basin in Brazil. The last cluster of seven cyprinodontiformids-infecting putative species of *Myxobolus* subclade VII (OMsCVII_species_1-7) showed close relationship to three *Myxobolus* spp. (MW589655, AY950664 and FJ468489) described from North American fishes (two cyprinodontiforms and a perciform).

In the polychaete-infecting lineage, *Ellipsomyxa papantla* sp. n. and two new putative species (PBTC_species_1 & 2) cluster with *Ellipsomyxa* spp. from Amazon basin freshwater fishes expanding the diversity of representatives of this genus. The other two new species (PBTC_species_3 & 4) revealed a new lineage of ellipsomyxids infecting the catadromous fish *Dajaus monticola*.

### Infection occurrence

Seven myxospore morphotypes detected in fishes are not formally described as new species herein due to an insufficient amount of data or to their occurrence as mixed infections. We report them with all available morphological data (Table 2; Fig. 3), as well as their sequence data (Supplementary Data 3) as a resource for future species investigations.

Apart from the four described species, 37 potential new taxa were inferred from the molecular and phylogenetic data. Most of the myxozoan species were detected in one single host species (31/41), while ten species showed no host specificity. Eight myxozoan species were detected in two different fish host species (OUTC_species_1- 3, *Zschokkella guelaguetza* sp. n., OMsCVII_species_5, OMsCVII_species_8, OMsCVII_species_17 and PBTC_species_1) and two myxozoans, *Myxidium zapotecus* sp. n. and a marine representative PBTC_species_2 were detected in three host species (Table 3).

The fish host species harboring the highest number of myxozoans was the mountain mullet *Dajaus monticola*, with eight species: six from oligochaete-infecting clade, including *Myxidium zapotecus* sp. n. in the biliary tract clade, *Myxobolus zoqueus* sp. n. and four others undescribed *Myxobolus* species from subclade VII, together with two from the polychaete-infecting biliary tract clade. We detected up to four species in the shortfin molly *Poecilia mexicana*: three species belonging to the *Myxobolus* subclade VII and one to the polychaete-infecting biliary tract clade. The fat sleeper *Dormitator maculatus* also harbored at least four species each in one of the following clades: oligochaete-infecting urinary tract, biliary tract, *Myxobolus* subclade VII and polychaete-infecting biliary tract clade, as well as the Oaxaca killifish *Profundulus punctatus*, with four species in the *Myxobolus* clade (one in subclade I, and three in subclade VII). Three myxozoan species were detected in the river goby *Awaous banana* (all three belonging to the oligochaete-infecting biliary tract, including *Myxidium zapotecus* sp. n. and *Zschokkella guelaguetza* sp. n.) and three in the Mexican mojarra *Mayaheros urophthalmus*, two belonging to *Myxobolus* subclade I and VII and one to the polychaete-infecting biliary tract clade (Supplementary Data 5).

Detection of mixed infections from gall bladder, meaning more than one species of myxozoan detected in a single fish specimen, occurred in 33.3% of the infected hosts (17 of 51). From those, 16 hosts harbor two taxa/species and only in one specimen of an unidentified *Paraneetroplus* sp. (Cichlidae), three species were detected from gall bladder. The most common combination was a representative of the polychaete-infecting biliary tract clade and one representative of the *Myxobolus* subclade VII (n = 5) or two representatives of the oligochaete-infecting biliary tract clade (n = 3) (Supplementary Data 3).

Detection of myxozoans by localities (with > 5 specimens collected), showed that three localities had the highest myxozoan prevalence: Río Negro, Santa María Chimalapa, Oaxaca (58.3%; 7/12), Río Los Perros, Santa María, Oaxaca (57.1%, 4/7) and Río La Palma, Veracruz (47.6%; 10/21). When calculating a discovery rate of species of myxozoans by number of fishes collected in that locality, three localities had the highest rates: Río Negro, Santa María Chimalapa, Oaxaca (0.7), Río la Palma, Veracruz (0.7) and Río Grande Matías Romero, Oaxaca (0.5) (Supplementary Data 6).

## Discussion

A large-scale evolutionary diversification of myxozoan species occurred after they acquired fishes as secondary hosts in their life cycles^28^. The highest concentration of freshwater fish diversity in the world occurs in Neotropical regions, with > 6200 species described and estimates of around 9000^38^. This suggests that high diversity of myxozoan species in this region could be expected. The parasite diversity detected in this study is probably just a scratch in the surface of the myxozoan fauna in the Neotropical region of Mexico. Myxozoan infections were detected in 90% of the freshwater fish species (17/19). From those, 65% had 1 or 2 myxozoan species and the remaining 35% had 3 or more species. This aligns with previous speculations of at least two novel myxozoan species per freshwater fish species in the Neotropical region^39^. We speculate that the number of species may be higher, as our study was limited to few organs, and screening of a wider range of organs and tissues may reveal a larger diversity in this region.

Geographically, several localities in Oaxaca had higher myxozoan prevalence than other localities in Veracruz and Chiapas, while overall, all localities in Veracruz had a higher myxozoan discovery rate per fish. However, neither locality or host species were sampled consistently, and the material collected was dependent on the catch success in each locality. Our results unfold lots of exciting questions about the distribution and diversity of these parasites, as for example, if certain river basins are more diverse than others or if differences exist between basins draining to the Pacific vs to the Atlantic. From the 536 species of freshwater fish reported in Mexico, more than half are found in the Nearctic region, of larger extension than the Neotropical area^40,41^. Exploring the diversity of myxozoans in this region and their comparison to Neotropical areas can provide useful information for diversity and conservation purposes; nearly 35% of the fish species are threatened or nearly threatened in Mexico^41^. Parasites play fundamental roles in the ecosystem dynamics and influence host evolutionary trajectories^42^. Populations of *Profundulus oaxacae* and *Rhamdia guatemalensis*, two fish hosts analyzed in this study, are a matter of concern and measurements of protection are being placed^43^. We detected three myxozoan species in these hosts. The risk of extinction of the host, is not only a risk for the fish but also for their associated parasites, which remain, in many cases, unknown.

The molecular screening allowed detection of myxozoans from fish tissue without a laborious microscopy evaluation, proving to be a useful approach for biodiversity exploration. This method increased the myxozoan prevalence detection up to 6 ×, representing 3.7 × more species detected by PCR screening than by microscopy; this represents nearly 1.3 × higher than a recent similar screening of fish tissues from the Czech Republic (2.8 ×). In this same study, a newly developed eDNA metabarcoding assay was shown to capture up to 2.5 × more diversity than PCR and Sanger sequencing in fish tissue^44^. The molecular screening carried in this study represents a useful tool for myxozoan diversity exploration and can be used for preliminary screening of fish tissue, representing a less intensive labor method than microscopy screening and a more accessible/less complex methodology than eDNA metabarcoding. However, eDNA metabarcoding assays of myxozoans may provide a closer to reality biodiversity estimation, especially when using fish tissues. Lisnerová et al.^44^ also demonstrated the effectivity of these assays when applied to sediment samples, as a non-invasive (no fish sacrifice) technique. This could represent an excellent alternative for future myxozoan diversity estimation in Mexico, where many fish species are rare, threatened or endangered.

Myxozoan proliferative stages and early sporogonic development can be easily detected using molecular techniques. Thus, myxozoan DNA detection does not imply that the parasites develops specifically in its tissues, as positive detection may result from the presence of proliferative stages in the vascular system^45^, or tissue contamination by species sporulating in the body cavity^46^. This may explain the high detection of *Myxobolus* spp. in this study. Their detection by PCR may suggest their presence as early blood stages or as sporulating in adjacent organs to the gall bladder. Future studies should determine the tissue specificity of these species in their hosts.

Several of the fish hosts in this study inhabit brackish waters or have migratory behavior, offering a high encounter opportunity to a wider range of invertebrates-infecting myxozoans. That is the case of the Mountain mullet *Dajaus monticola* who seems to have remarkable diversity of myxozoans (8 species). Mugilids are known hosts for myxozoans, with high diversity reported in other regions of the world^47^. The Mountain mullet is a catadromous species, a characteristic that could explain the abundance of myxozoan species and their assembly composition, with oligochaete and polychaete-infecting myxozoan representatives of three different clades. This species migration and its presence in brackish water could represent a great opportunity for myxozoan dispersion, colonization and transitioning to other environments.

Most of the host species analyzed in this study are of commercial economic importance for fisheries, gaming and/or aquaculture in Mexico^48^ (FishBase—Supplementary Data 1). We detected several myxozoan infections in commercially valuable ornamental fishes for aquarium trade like poecilids: shortfin molly *Poecilia mexicana* (4 species), molly *Poecilia sphenops* (1 species), the Chiapas swordtail *Xiphophorus alvarezi* (2 species), or as fish food for aquaculture, like the Mexican mojarra *Mayaheros urophtalmus* (3 species) and the South American catfish *Rhamdia quelen* (2 species). The myxozoan diversity unraveled in this study represents a source of emerging disease agents, that could arise under environmental change or under farming conditions^49^. Most of the commercial trade of these species is still heavily dependent on wild populations^50^, which may be carriers of myxozoan infections into culture/rearing facilities. These infections may not be harmful in natural conditions but can become a health issue under stressful culture conditions, as either primary pathogens, like *Myxobolus* spp.^51^ or opportunistic ones, like the ones found in the hepatic-biliary system of fishes^52^. Our results suggest that fish facilities should include myxozoans in their parasitological screenings and monitor fish health as these parasites could become a cause for concern.

Aquarium fishes are a large reservoir of invasive species. The exportation of native fishes as pets have resulted in biological introductions in other regions of the world. For example, *Poecilia mexicana* can been found in Oceania, *Poecilia sphenops* in Asia and Europe or *Mayaheros urophtalmus* in Asia (FishBase), which are some of the hosts analysed in this study. This not only represents a biological invasion that threatens native fish populations but also a biosecurity risk by the co-introduction of their native myxozoan infections^49^. Recently, myxozoan infections were detected in ornamental fish imported from Asia to Australia, which were previously undetected in sanitary inspections^53^. This lack of detection seems to be a common situation, as witnessed by the few published surveys about myxozoans in aquatic pet animals^49^.

In contrast, establishment of non-native fish farms in Mexico is widespread due to the lack of regulations^54^. Some of these species are already invasive, while others were identified as high-risk invasiveness. These exotic fishes can represent new hosts for the native parasites, amongst them myxozoans. While myxozoans have complex life cycles and certain requisites have to be accomplished for the establishment of a myxozoan infection i.e., presence of adequate vertebrate and invertebrate host, temperature-dependent development, longevity of waterborne stages, and others^49^, introductions of myxozoans have occurred in some cases with devastating consequences (*e.g. Myxobolus cerebralis*^55^, *Kudoa iwatai*^56^, *Myxobolus dechtiari*^57^).

## Conclusions

Myxozoans represent a neglected and understudied group of parasites in Mexico. This study demonstrates that the highly diverse group of Neotropical fishes are hosts of a multitude of myxozoan species and that they represent an interesting research area for conservation and economic reasons in Mexico. Fish in this country are commercialized for food or for aquarium trade, including international trade; several are, due to human activities, under risk or already threatened species. In order to design strategies to limit myxozoan introductions and outbreaks, it is essential to increase our knowledge on the baseline biodiversity and taxonomic data to identify emerging myxozoans diseases. Future studies should include non-invasive environmental DNA metabarcoding to fully comprehend the diversity of myxozoan species in Mexico. We hope this manuscript will promote research on this fascinating group of parasitic cnidarians in the megadiverse country of Mexico.

## Supplementary Information

Supplementary Information 1.

Supplementary Information 2.

Supplementary Information 3.

Supplementary Information 4.

Supplementary Information 5.

Supplementary Information 6.

## Data Availability

Archival smears of described species are deposited at the Institute of Biology (Universidad Nacional Autónoma de México, UNAM), Colección Nacional de Helmintos CNHE 11950-11953. The datasets generated and/or analyzed during the current study are available in GenBank repository, Acc. Number OQ888222-OQ888300.

## References

[1] Feist S, Longshaw M, Woo PTK (2006). Phylum Myxozoa. Fish Diseases and Disorders Protozoan and Metazoan Infections.

[2] Zhang Z (2013). Animal biodiversity: An update of classification and diversity in 2013. Zootaxa.

[3] Okamura B, Gruhl A, Bartholomew JL, Okamura B, Gruhl A, Bartholomew J (2015). An introduction to myxozoan evolution, ecology and development. Myxozoan Evolution, Ecology and Development.

[4] Appeltans W (2012). The magnitude of global marine species diversity. Curr. Biol..

[5] Myers N, Mittermeier RA, Mittermeier CG, da Fonseca GA, Kent J (2000). Biodiversity hotspots for conservation priorities. Nature.

[6] Scholz T, Choudhury A (2014). Parasites of freshwater fishes in North America: Why so neglected?. J. Parasitol..

[7] Choudhury A, García-Varela M, Pérez-Ponce de León G (2017). Parasites of freshwater fishes and the Great American Biotic Interchange: A bridge too far*?*. J. Helminthol..

[8] Noble ER (1966). Myxosporida in deepwater fishes. J. Parasitol..

[9] Hoffman GL, Putz RE, Dunbar CE (1965). Studies on *Myxosoma cartilaginis* n. sp. (Protozoa: Myxosporidea) of Centrarchid fish and a Synopsis of the *Myxosoma* of North American Freshwater Fishes. J. Protozool..

[10] Segovia-Salinas, F. & Jiménez-Guzmán, F. *Myxobolus cartilaginis* (Myxosporea: Myxobolidae) parasite of *Micropterus salmoides* at the Rodrigo Gomez dam, Nuevo Leon, Mexico. Publicaciones Biologicas F.C.B/U.A.N.L., Mexico, **5**, 70–72 (1991).

[11] Segovia-Salinas S, Jiménez-Guzmán FE, Galaviz-Silva L, Ramírez-Bom E (1991). *Myxobolus nuevoleonsis* n. sp. (Myxozoa: Myxobolidae) parasite of fishes *Poecilia mexicana* and *P. reticulata* from Rio de la Silla near Monterrey, Nuevo Leon, Mexico. Rev. Latinoam. Microbiol..

[12] Rábago-Castro JL (2013). Spatial and seasonal variations on *Henneguya exilis* prevalence on cage intensive cultured channel catfish (*Ictalurus punctatus*), in Tamaulipas, Mexico. Lat. Am. J. Aquat. Res..

[13] Dyková I, Avila EJF, Fiala I (2002). *Kudoa dianae* sp. N. (Myxosporea: Multivalvulida) a new parasite of bullseye puffer, *Sphoeroides annulatus* (Tetraodontiformes: Tetraodontidae). Folia Parasitol..

[14] Vidal LP, Iannacone J, Whipps CM, Luque JL (2017). Synopsis of the species of Myxozoa Grassé, 1970 (Cnidaria: Myxosporea) in the Americas. Neotrop. Helminthol..

[15] Fiala I (2006). The phylogeny of Myxosporea (Myxozoa) based on small subunit ribosomal RNA gene analysis. Int. J. Parasitol..

[16] Auró de Ocampo A, Ocampo Camberos L (1998). Caracterización histopatológica de la respuesta de la tilapia *Oreochromis* sp. a una infección mixta por myxosporidios Estudio en un caso natural. Vet. Mex..

[17] Miller RR, Minckley WL, Norris SM (2006). Freshwater Fishes of Mexico.

[18] Říčan O, Piálek L, Dragová K, Novák J (2016). Diversity and evolution of the Middle American cichlid fishes (Teleostei: Cichlidae) with revised classification. Vertebr. Zool..

[19] Froese, R. & Pauly, D. FishBase. World Wide Web electronic publication. www.fishbase.org, version (08/2022).

[20] Lom J, Arthur JR (1989). A guideline for the preparation of species descriptions in Myxosporea. J. Fish Dis..

[21] Ben-David J (2016). Myxozoan polar tubules display structural and functional variation. Parasit. Vectors..

[22] Schneider CA, Rasband WS, Eliceiri KW (2012). NIH Image to ImageJ: 25 years of image analysis. Nat. Methods..

[23] Alama-Bermejo G, Bron JE, Raga JA, Holzer AS (2012). 3D Morphology, ultrastructure and development of *Ceratomyxa puntazzi* stages: first insights into the mechanisms of motility and budding in the Myxozoa. PLoS ONE.

[24] Asahida T, Kobayashi T, Saitoh K, Nakayama I (1996). Tissue preservation and total DNA extraction from fish stored at ambient temperature using buffers containing high concentration of urea. Fish. Sci..

[25] Barta JR (1997). Phylogenetic relationships among eight *Eimeria* species infecting domestic fowl inferred using complete small subunit ribosomal DNA sequences. J. Parasitol..

[26] Kent ML, Khattra J, Hervio DML, Devlin RH (1998). Ribosomal DNA sequence analysis of isolates of the PKX myxosporean and their relationship to members of the genus *Sphaerospora*. J. Aquat. Anim. Health..

[27] Hallett SL, Diamant A (2001). Ultrastructure and small-subunit ribosomal DNA sequence of *Henneguya lesteri* n. sp. (Myxosporea), a parasite of sand whiting *Sillago analis* (Sillaginidae) from the coast of Queensland, Australia. Dis. Aquat. Org..

[28] Holzer AS (2018). The joint evolution of the Myxozoa and their alternate hosts: A cnidarian recipe for success and vast biodiversity. Mol Ecol..

[29] Katoh K, Standley DM (2013). MAFFT multiple sequence alignment software version 7: Improvements in performance and usability. Mol. Biol. Evol..

[30] Stamatakis A (2014). RAxML version 8: a tool for phylogenetic analysis and post-analysis of large phylogenies. Bioinformatics.

[31] Darriba D, Taboada GL, Doallo R, Posada D (2012). jModelTest 2: more models, new heuristics and parallel computing. Nat. Methods..

[32] Ronquist F, Huelsenbeck JP (2003). MrBayes 3: Bayesian phylogenetic inference under mixed models. Bioinformatics.

[33] Rambaut A, Drummond AJ, Xie D, Baele G, Suchard MA (2018). Posterior summarisation in Bayesian phylogenetics using Tracer 1.7. Syst. Biol..

[34] Eiras JC, Saraiva A, Cruz CF, Santos MJ, Fiala I (2011). Synopsis of the species of *Myxidium* Bütschli, 1882 (Myxozoa: Myxosporea: Bivalvulida). Syst. Parasitol..

[35] Matsche MA, Yurakhno V, Zhang J, Sato H (2021). Synopsis of the species of the genus *Zschokkella* Auerbach, 1910 (Myxozoa: Bivalvulida: Myxidiidae). Syst. Parasitol..

[36] Yoshino TP, Noble ER (1973). Myxosporida in macrourid fishes of the North Atlantic. Can. J. Zool..

[37] Liu Y, Lövy A, Gu ZM, Fiala I (2019). Phylogeny of Myxobolidae (Myxozoa) and the evolution of myxospore appendages in the *Myxobolus* clade. Int. J. Parasitol..

[38] Albert JS, Tagliacollo VA, Dagosta F (2020). Diversification of neotropical freshwater fishes. Annu. Rev. Ecol. Evol. Syst..

[39] Naldoni J (2011). Host-parasite-environment relationship, morphology and molecular analyses of *Henneguya eirasi* n. sp. parasite of two wild *Pseudoplatystoma* spp. in Pantanal Wetland, Brazil. Vet. Parasitol..

[40] Torres-Orozco Bermeo, R. E. & Pérez Hernández, M. A. Riqueza y regionalización de los peces de México. Ciencia - Academia Mexicana de Ciencias, México, **60(**3), 44–53 (2009).

[41] Lyons, T. J. *et al.* The status and distribution of freshwater fishes in Mexico. Cambridge, UK and Albuquerque, New Mexico, USA: IUCN and ABQ BioPark, 80 p. (2020).

[42] Okamura B, Hartigan A, Naldoni J (2018). Extensive uncharted biodiversity: The parasite dimension. Integr. Comp. Biol..

[43] Martínez-Ramírez, E., Doadrio, I. & Sostoa-Fernández, A. Peces Continentales. Biodiversidad de Oaxaca, 357–373 (2004).

[44] Lisnerová M, Holzer A, Blabolil P, Fiala I (2023). Evaluation and optimization of an eDNA metabarcoding assay for detection of freshwater myxozoan communities. Environ. DNA..

[45] Holzer AS, Hartigan A, Patra S, Pecková H, Eszterbauer E (2014). Molecular fingerprinting of the myxozoan community in common carp suffering swim bladder inflammation (SBI) identifies multiple etiological agents. Parasit. Vectors.

[46] Liu XH (2016). Morphological and molecular characterisation of *Myxobolus pronini* n. sp. (Myxozoa: Myxobolidae) from the abdominal cavity and visceral serous membranes of the gibel carp *Carassius auratus* gibelio (Bloch) in Russia and China. Parasit. Vectors.

[47] Rocha S (2019). Myxozoan biodiversity in mullets (Teleostei, Mugilidae) unravels hyperdiversification of *Myxobolus* (Cnidaria, Myxosporea*)*. Parasitol. Res..

[48] Dávila-Camacho CA (2019). Cultivation of native fish in Mexico: Cases of success. Rev. Aquac..

[49] Hallett SL, Hartigan A, Atkinson SD, Okamura B, Gruhl A, Bartholomew J (2015). Myxozoans on the move: Dispersal modes, exotic species and emerging diseases. Myxozoan Evolution, Ecology and Development.

[50] Teletchea F (2016). Domestication level of the most popular aquarium fish species: Is the aquarium trade dependent on wild populations?. Cybium.

[51] Sarker S, Kallert DM, Hedrick RP, El-Matbouli M (2015). Whirling disease revisited: Pathogenesis, parasite biology and disease intervention. Dis. Aquat. Org..

[52] Katharios P, Garaffo M, Sarter K, Athanassopoulou F, Mylonas CC (2007). A case of high mortality due to heavy infestation of *Ceratomyxa diplodae* in sharpsnout sea bream (*Diplodus puntazzo*) treated with reproductive steroids. Bull. Eur. Ass. Fish Pathol..

[53] Trujillo-González A, Allas J, Miller TL, Becker JA, Hutson KS (2022). Myxozoan diversity infecting ornamental fishes imported to Australia. Front. Mar. Sci..

[54] Mendoza R, Luna S, Aguilera C (2015). Risk assessment of the ornamental fish trade in Mexico: Analysis of freshwater species and effectiveness of the FISK (Fish Invasiveness Screening Kit). Biol. Invasions..

[55] Bartholomew JL, Reno PW, Bartholomew JL, Wilson JC (2002). The history of the dissemination of whirling disease. Whirling Disease Reviews. American Fisheries Society Symposium 26.

[56] Diamant A, Ucko M, Paperna I, Colorni A, Lipshitz A (2005). *Kudoa iwatai* (Myxosporea: Multivalvulida) in wild and cultured fish in the Red Sea: re-description and molecular phylogeny. J. Parasitol..

[57] Goswami U (2021). Evidence of the American *Myxobolus dechtiari* was introduced along with its host *Lepomis gibbosus* in Europe: Molecular and histological data. Int. J. Parasitol. Parasites Wildl..

