# Supplementary material for: Diversity of myxozoans (Cnidaria) infecting Neotropical fishes in southern Mexico
Source: Sci Rep. 2023 Jul 26;13:12106. doi: 10.1038/s41598-023-38482-2 (PMC10372099; doi:10.1038/s41598-023-38482-2)
Supplement: Supplementary file 1 — Supplementary Information 1. [file 41598_2023_38482_MOESM1_ESM.docx]

**Supplementary Data 1:** Biological and geographical data of the fish species examined in this study. Fish total length (TL) in millimeters. Collection date in format: Year.Month.Day. Information on the habitat and occurrence of fish is restricted to Mexico. Data on economic value of each fish species retrieved from FishBase (www.fishbase.org).

| **Host** | **n** | **TL** | **Collection date** | **Locality** | **Coordinates** | **Habitat** | **Occurrence** | **Human uses** |
| --- | --- | --- | --- | --- | --- | --- | --- | --- |
| **Characiformes: Characidae** |  |  |  |  |  |  |  |  |
| *Astyanax* sp. | 3 | 68–105 | 2014.03.23 | Río Negro, Santa María Chimalapa, Oaxaca | 16°53'55''N; 94°41'37''W | -- | -- | -- |
| **Cyprinodontiformes: Poeciliidae** |  |  |  |  |  |  |  |  |
| *Poecilia mexicana* Steindachner | 4 | 75–80 | 2014.03.18 | Río La Palma, Veracruz | 18º33'21''N; 95º2'59''W | Freshwater | Native | Aquarium: highly commercial |
|  | 1 | 56 | 2014.03.19 | Tlacotalpan, Veracruz | 18º36'41''N; 95º39'44''W |  |  |  |
|  | 3 | 96–112 | 2014.03.22 | Río La Palma, Veracruz | 18º33'21''N; 95º2'59''W |  |  |  |
| *Poecilia sphenops* Valenciennes | 5 | 61–72 | 2014.06.07 | Tlacotalpan, Veracruz | 18º36'41''N; 95º39'44''W | Freshwater, Brackish | Native | Aquarium: commercial |
|  | 1 | 95 | 2015.03.26 | Santa María, Guienagati, Oaxaca | 16°44'22"N; 95°21'23"W |  |  |  |
| *Xiphophorus alvarezi* Rosen | 4 | 60–82 | 2014.03.18 | Río La Palma, Veracruz | 18º33'21''N; 95º2'59''W | Freshwater | Native | Aquarium: commercial |
| *Xiphophorus* sp. | 1 | 63 | 2014.03.22 | Río La Palma, Veracruz | 18º33'21''N; 95º2'59''W | -- | -- | -- |
| **Cyprinodontiformes: Profundulidae** |  |  |  |  |  |  |  |  |
| *Tlaloc labialis* (Günther) | 1 | 84 | 2014.03.24 | Río San Juan, Cristobal Obregón, Chiapas | 16°21'00''N; 93°30'54''W | Freshwater | Native | Aquarium: commercial |
| *Profundulus oaxacae* (Meek) | 7 | 51–73 | 2015.03.29 | Río los Sabinos, Oaxaca | 15°55'50"N; 95°55'58"W | Freshwater | Native | -- |
|  | 5 | 57–78 | 2015.03.29 | El Toronjo, Oaxaca | 16°26'15"N; 97° 3'50"W |  |  |  |
|  | 2 | 54–63 | 2015.03.30 | Río Grande, Mitla, Oaxaca | 16°55'41"N; 96°19'32"W |  |  |  |
| *Profundulus punctatus* (Günther) | 2 | 66–72 | 2014.03.24 | Río San Juan, Cristóbal Obregón, Chiapas | 16°21'00''N; 93°30'54''W | Freshwater | Native | Aquarium: commercial |
|  | 7 | 58–71 | 2015.03.26 | Río los Perros, Santa María, Oaxaca | 16°44'23"N; 95°21'23"W |  |  |  |
|  | 3 | 48–64 | 2014.03.27 | Río Huixtla, Chiapas | 15°10'18''N; 92°25'24''W |  |  |  |
|  | 1 | 53 | 2014.03.27 | Nueva Francia, Chiapas | 15°22'08''N; 92°35'20''W |  |  |  |
|  | 5 | 59–76 | 2014.03.27 | El Triunfo, Chiapas | 15°20'44''N; 92°32'30''W |  |  |  |
|  | 5 | 59–68 | 2015.03.28 | Río Chacalapa, Oaxaca | 15°55'50"N; 95°55'58"W |  |  |  |
| **Mugiliformes: Mugilidae** |  |  |  |  |  |  |  |  |
| *Dajaus monticola* (Bancroft) | 4 | 77–131 | 2014.03.18 | Río La Palma, Veracruz | 18º33'21''N; 95º2'59''W | Brackish, Freshwater | Native | Fisheries: subsistence |
|  | 3 | 95–119 | 2015.03.26 | Rio Grande, Matías Romero, Oaxaca | 16°47'29"N; 95°01'02"W |  |  |  |
| **Perciformes: Cichlidae** |  |  |  |  |  |  |  |  |
| *Cichlasoma trimaculatum* (Günther) | 1 | 135 | 2015.03.26 | Río Tequisistlán, Oaxaca | 16°24'52"N; 95°35'58"W | Freshwater | Native | Aquarium: commercial |
| *Mayaheros urophthalmus* (Günther) | 2 | 110–150 | 2014.03.19 | Tlacotalpan, Veracruz | 18º36'41''N; 95º39'44''W | Freshwater, Brackish | Native, Threatened | Fisheries: commercial; aquaculture: commercial; gamefish: yes; aquarium: commercial |
|  | 2 | 136–143 | 2015.03.21 | Tlacotalpan, Veracruz | 18º36'41''N; 95º39'44''W |  |  |  |
| *Maskaheros regani* (Miller) | 1 | 184 | 2015.03.25 | Río Grande, Matías Romero, Oaxaca | 16°47'29"N; 95°01'02"W | Freshwater | Endemic | -- |
| *Parachromis friedrichsthalii* (Heckel) | 4 | 163–176 | 2015.03.26 | Río Grande, Matías Romero, Oaxaca | 16°47'29"N; 95°01'02"W | Freshwater, Brackish | Native | Gamefish: yes; aquarium: commercial |
| *Paraneetroplus bulleri* Regan | 2 | 98–114 | 2014.03.23 | Río Negro, Santa María Chimalapa, Oaxaca | 16°53'55''N; 94°41'37''W | Freshwater | Endemic | -- |
|  | 1 | 142 | 2015.03.26 | Río Grande, Matías Romero, Oaxaca | 16°47'29"N; 95°01'02"W |  |  |  |
| *Paraneetroplus* sp. | 2 | 74–212 | 2014.03.23 | Río Negro, Santa María Chimalapa, Oaxaca | 16°53'55''N; 94°41'37''W | -- | -- | -- |
| *Thorichthys maculipinnis* (Steindachner) | 2 | 76–83 | 2015.03.25 | Río Grande, Matías Romero, Oaxaca | 16°47'29"N; 95°01'02"W | Freshwater | Endemic | -- |
| *Thorichthys* sp. | 2 | 76–81 | 2014.03.23 | Río Negro, Santa María Chimalapa, Oaxaca | 16°53'55''N; 94°41'37''W | -- | -- | -- |
| *Vieja fenestrata* (Günther) | 2 | 163–179 | 2015.03.25 | Río Grande, Matías Romero, Oaxaca | 16°47'29"N; 95°01'02"W | Freshwater | Endemic | -- |
| *Vieja zonata* (Meek) | 1 | 115 | 2015.03.26 | Río Tequisistlán, Oaxaca | 16°24'52"N; 95°35'57"W | Freshwater | Endemic | -- |
| **Perciformes: Eleotridae** |  |  |  |  |  |  |  |  |
| *Dormitator maculatus* (Bloch) | 6 | 87–122 | 2014.03.19 | Tlacotalpan, Veracruz | 18º36'41''N; 95º39'44''W | Saltwater | Native | Aquarium: commercial |
|  | 5 | 106–128 | 2015.03.21 | Tlacotalpan, Veracruz | 18º36'41''N; 95º39'44''W |  |  |  |
| *Gobiomorus dormitor* Lacepède | 2 | 35–42 | 2014.03.18 | Río La Palma, Veracruz | 18º33'21''N; 95º2'59''W | Saltwater | Native | Fisheries |
| *Eleotris* sp. | 2 | 115–123 | 2014.03.18 | Río La Palma, Veracruz | 18º33'21''N; 95º2'59''W | -- | -- | -- |
| **Perciformes: Gobiidae** |  |  |  |  |  |  |  |  |
| *Awaous banana* (Valenciennes) | 1 | 213 | 2014.03.23 | Río Negro, Santa María Chimalapa, Oaxaca | 16°53'55''N; 94°41'37''W | Freshwater | Native | Fisheries: commercial |
|  | 2 | 193–203 | 2015.03.26 | Río Grande, Matías Romero, Oaxaca | 16°47'29"N; 95°01'02"W |  |  |  |
| **Perciformes: Scombridae** |  |  |  |  |  |  |  |  |
| *Sarda sarda* (Bloch) | 1 | 580 | 2014.06.12 | Alvarado, Veracruz | 18°46'24''N; 95°45'00''W | Saltwater, Brackish | Native | Fisheries: highly commercial; gamefish: yes |
| **Siluriformes: Heptapteridae** |  |  |  |  |  |  |  |  |
| *Rhamdia quelen* (Quoy & Gaimard) | 1 | 93 | 2014.03.23 | Río Negro, Santa María Chimalapa, Oaxaca | 16°53'55''N; 94°41'37''W | Freshwater | Native | Fisheries: minor commercial; aquaculture: commercial |
|  | 5 | 73–124 | 2014.03.24 | Río San Juan, Cristobal Obregón, Chiapas | 16°21'00''N; 93°30'54''W |  |  |  |
| *Rhamdia guatemalensis* (Günther) | 1 | 116 | 2015.03.24 | Catemaco, Veracruz | 18°25'00''N; 95°07'00''W | Freshwater | Native | -- |
|  | 2 | 76–88 | 2015.03.26 | Río Grande, Matías Romero, Oaxaca | 16°47'29"N; 95°01'02"W |  |  |  |
|  | 1 | 94 | 2015.03.26 | Santa María, Guienagati, Oaxaca | 16°44'23"N; 95°21'23"W |  |  |  |
| **Synbranchiformes: Synbranchidae** |  |  |  |  |  |  |  |  |
| *Synbranchus marmoratus* Bloch | 1 | 325 | 2014.03.23 | Río Negro, Santa María Chimalapa, Oaxaca | 16°53'55''N; 94°41'37''W | Brackish, Freshwater | Native | Fisheries: commercial; aquarium: public aquariums |
|  | 1 | 73 | 2014.03.27 | Río Huixtla, Chiapas | 15°10'18''N; 92°25'24''W |  |  |  |
| Total fish | 120 |  |  |  |  |  |  |  |
