# Supplementary material for: Diversity of myxozoans (Cnidaria) infecting Neotropical fishes in southern Mexico
Source: Sci Rep. 2023 Jul 26;13:12106. doi: 10.1038/s41598-023-38482-2 (PMC10372099; doi:10.1038/s41598-023-38482-2)
Supplement: Supplementary file 2 — Supplementary Information 2. [file 41598_2023_38482_MOESM2_ESM.docx]

**Supplementary Data 2.** Primer combinations applied to amplify longer fragments of the SSU rDNA region of selected samples. PCR conditions were as described in the methods section.

| **Species** | **Sample code** | **Primer name** | **Sequence (5’-3’)** | **Reference** | **PCR combinations** | **Total length sequence (bp)** |
| --- | --- | --- | --- | --- | --- | --- |
| *Myxidium zapotecus* sp. n. | C8 | ERIB1 | ACC TGG TTG ATC CTG CCA G | Barta et al. (1997) | 1) ERIB1-ERIB10 and nested Myxgp2f- ACT1r  2) ERIB1- ACT1r  3) Myxgen4F-ERIB10  4) MyxospecF-ERIB10 | 1,806 |
|  |  | ERIB10 | CTT CCG CAG GTT CAC CTA CGG | Barta et al. (1997) |  |  |
|  |  | Myxgp2f | WTG GAT AAC CGT GGG AAA | Kent et al. (1998) |  |  |
|  |  | ACT1r | AAT TTC ACC TCT CGC TGC CA | Hallett & Diamant, (2001) |  |  |
|  |  | Myxgen4F | GTG CCT TGA ATA AAT CAG AG | Diamant et al. (2004) |  |  |
|  |  | MyxospecF | TTC TGC CCT ATC AAC TWG TTG | Fiala (2006) |  |  |
| *Zschokkella guelaguetza* sp. n. | N26 | ERIB1 | ACC TGG TTG ATC CTG CCA G | Barta et al. (1997) | 1) ERIB1-ERIB10 and nested Myxgp2f- ACT1r  2) ERIB1- ACT1r  3) Myxgen4F-ERIB10  4) MyxospecF-ERIB10 | 1,926 |
|  |  | ERIB10 | CTT CCG CAG GTT CAC CTA CGG | Barta et al. (1997) |  |  |
|  |  | Myxgp2f | WTG GAT AAC CGT GGG AAA | Kent et al. (1998) |  |  |
|  |  | ACT1r | AAT TTC ACC TCT CGC TGC CA | Hallett & Diamant, (2001) |  |  |
|  |  | Myxgen4F | GTG CCT TGA ATA AAT CAG AG | Diamant et al. (2004) |  |  |
|  |  | MyxospecF | TTC TGC CCT ATC AAC TWG TTG | Fiala (2006) |  |  |
| *Zschokkella* sp. | L4 | ERIB1 | ACC TGG TTG ATC CTG CCA G | Barta et al. (1997) | 1) ERIB1-ERIB10 and nested Myxgp2f- ACT1r  2) ERIB1- ACT1r  3) Myxgen4F-ERIB10 | 1,963 |
|  |  | ERIB10 | CTT CCG CAG GTT CAC CTA CGG | Barta et al. (1997) |  |  |
|  |  | Myxgp2f | WTG GAT AAC CGT GGG AAA | Kent et al. (1998) |  |  |
|  |  | ACT1r | AAT TTC ACC TCT CGC TGC CA | Hallett & Diamant, (2001) |  |  |
|  |  | Myxgen4F | GTG CCT TGA ATA AAT CAG AG | Diamant et al. (2004) |  |  |
| *Ellipsomyxa papantla* sp. n. | N4 | ERIB1 | ACC TGG TTG ATC CTG CCA G | Barta et al. (1997) | 1) ERIB1-ERIB10 and nested Myxgp2f- ACT1r  2) ERIB1- ACT1r  3)MyxospecF-18R (followed by cloning and sequencing with M13R) | 1,603 |
|  |  | ERIB10 | CTT CCG CAG GTT CAC CTA CGG | Barta et al. (1997) |  |  |
|  |  | Myxgp2f | WTG GAT AAC CGT GGG AAA | Kent et al. (1998) |  |  |
|  |  | ACT1r | AAT TTC ACC TCT CGC TGC CA | Hallett & Diamant, (2001) |  |  |
|  |  | MyxospecF | TTC TGC CCT ATC AAC TWG TTG | Fiala (2006) |  |  |
|  |  | 18R | CTA CGG AAA CCT TGT TAC G | Whipps et al. (2003) |  |  |
| *Myxobolus zoqueus* sp. n. | N21 | ERIB1 | ACC TGG TTG ATC CTG CCA G | Barta et al. (1997) | 1) ERIB1-ERIB10 and nested Myxgp2f- ACT1r  2) ERIB1- ACT1r  3) Myxgen4F-ERIB10 | 1,804 |
|  |  | ERIB10 | CTT CCG CAG GTT CAC CTA CGG | Barta et al. (1997) |  |  |
|  |  | Myxgp2f | WTG GAT AAC CGT GGG AAA | Kent et al. (1998) |  |  |
|  |  | ACT1r | AAT TTC ACC TCT CGC TGC CA | Hallett & Diamant, (2001) |  |  |
|  |  | Myxgen4F | GTG CCT TGA ATA AAT CAG AG | Diamant et al. (2004) |  |  |

**Primers references**

Diamant A, Whipps CM, Kent ML. 2004. A new species of *Sphaeromyxa* (Myxosporea: Sphaeromyxina: Sphaeromyxidae) in devil firefish, *Pterois miles* (Scorpaenidae), from the northern Red Sea: morphology, ultrastructure, and phylogeny. Journal of Parasitology, *90*, 1434-1442.

Fiala I. 2006. The phylogeny of Myxosporea (Myxozoa) based on small subunit ribosomal RNA gene analysis. International Journal for Parasitology, 36, 1521-1534.

Kent ML, Khattra J, Hervio DML, Devlin RH. 1998. Ribosomal DNA sequence analysis of isolates of the PKX myxosporean and their relationship to members of the genus *Sphaerospora.* Journal of Aquatic Animal Health, 10, 12-21.

Barta JR, Martin DS, Liberator PA, Dashkevicz M, Anderson JW, Feighner SD, Elbrecht A, Perkins-Barrow A, Jenkins MC, Danforth HD, Ruff MD, Profous-Juchelka H. 1997. Phylogenetic relationships among eight *Eimeria* species infecting domestic fowl inferred using complete small subunit ribosomal DNA sequences. Journal of Parasitology, 83, 262-271.

Hallett SL, Diamant A. 2001. Ultrastructure and small-subunit ribosomal DNA sequence of *Henneguya lesteri* n. sp. (Myxosporea), a parasite of sand whiting *Sillago analis* (Sillaginidae) from the coast of Queensland, Australia. Diseases of aquatic organisms, 46, 197-212.

Whipps CM, Adlard RD, Bryant MS, Lester RJG, Findlay V, Kent ML. 2003. First report of three *Kudoa* species from eastern Australia: *Kudoa thyrsites* from mahi mahi (*Coryphaena hippurus*), *Kudoa amamiensis* and *Kudoa minithyrsites* n. sp. from sweeper (*Pempheris ypsilychnus*). Journal of Eukaryotic Microbiology, 50, 215-219.
