# Supplementary material for: Diversity of myxozoans (Cnidaria) infecting Neotropical fishes in southern Mexico
Source: Sci Rep. 2023 Jul 26;13:12106. doi: 10.1038/s41598-023-38482-2 (PMC10372099; doi:10.1038/s41598-023-38482-2)
Supplement: Supplementary file 5 — Supplementary Information 5. [file 41598_2023_38482_MOESM5_ESM.docx]

**Supplementary Data 5. –** Summary of number of sequences per host and locality and number of myxozoan species (new or putative) detected by locality and by fish host species. In bold species detected in more than one host species.

| **Fish species** | **Locality, number of fish and year** | **# Sequences** | **# species/host and locality** | **Species detected** | **# species/ host** | **Species detected** |
| --- | --- | --- | --- | --- | --- | --- |
| *Profundulus punctatus* (n=23) | Río San Juan, Cristobal Obregón, Chiapas (n=2) 2014 | - | - | - | 4 | OMsCI_species_1  OMsCVII_species_3  OMsCVII_species_14  **OMsCVII_species_17** |
|  | Río Huixtla, Chiapas (n=3) 2014 | 2 | 2 | OMsCI_species_1  OMsCVII_species_17 |  |  |
|  | Nueva Francia, Chiapas (n=1) 2014 | 1 | 1 | OMsCVII_species_3 |  |  |
|  | El Triunfo, Chiapas (n=5) 2014 | 3 | 2 | OMsCI_species_1  OMsCVII_species_3 |  |  |
|  | Río los Perros, Santa María, Oaxaca (n=7) 2015 | 4 | 2 | OMsCVII_species_3  OMsCVII_species_14 |  |  |
|  | Río Chacalapa, Oaxaca (n=5) 2015 | - | - | - |  |  |
| *Profundulus oaxacae* (n=14) | Río Los Sabinos, Oaxaca (n=7) 2015 | 3 | 2 | OMsCI_species_2  OMsCVII_species_4 | 2 | OMsCI_species_2  OMsCVII_species_4 |
|  | El Toronjo, Oaxaca (n=5) 2015 | - | - | - |  |  |
|  | Río Grande, Mitla, Oaxaca (n=2) 2015 | - | - | - |  |  |
| *Tlaloc labialis* (n=1) | Río San Juan, Cristobal Obregón, Chiapas (n=1) 2014 | - | - | - | - | - |
| *Poecilia mexicana* (n=8) | Río La Palma, Veracruz (n=7) 2014 | 4 | 3 | OMsCVII_species_1  OMsCVII_species_5  PBTC_species_2 | 4 | OMsCVII_species_1  OMsCVII_species_2  **OMsCVII_species_5**  **PBTC_species_2** |
|  | Tlacotalpan, Veracruz (n=1) 2014 | 1 | 1 | OMsCVII_species_2 |  |  |
| *Poecilia sphenops* (n=6) | Tlacotalpan, Veracruz (n=5) 2014 | 1 | 1 | OMsCVII_species_7 | 1 | OMsCVII_species_7 |
|  | Santa Maria, Guienagati, Oaxaca (n=1) 2015 | - | - | - |  |  |
| *Xiphophorus alvarezi* (n=4) | Río La Palma, Veracruz (n=4) 2014 | 4 | 2 | OMsCVII_species_12  PBTC_species_2 | 2 | OMsCVII_species_12  **PBTC_species_2** |
| *Dajaus monticola* (n=7) | Río La Palma, Veracruz (n=4) 2014 | 7 | 6 | OMsCVII_species_9  OMsCVII_species_10  OMsCVII_species_11  OMsCVII_species_13  PBTC_species_3  PBTC_species_4 | 8 | OMsCVII_species_9  OMsCVII_species_10  OMsCVII_species_11  OMsCVII_species_13  PBTC_species_3  PBTC_species_4  ***Myxidium zapotecus* sp. n.**  *Myxobolus zoqueus* sp. n. |
|  | Rio Grande, Matías Romero, Oaxaca (n=3) 2015 | 3 | 2 | *Myxidium zapotecus* sp. n.  *Myxobolus zoqueus* sp. n. |  |  |
| *Mayaheros urophthalmus* (n=4) | Tlacotalpan, Veracruz (n=2) 2014 | 1 | 1 | PBTC_species_1 | 3 | **PBTC_species_1**  OMsCI_species_3  **OMsCVII_species_8** |
|  | Tlacotalpan, Veracruz (n=2) 2015 | 2 | 2 | OMsCI_species_3  OMsCVII_species_8 |  |  |
| *Parachromis friedrichsthalii* (n=4) | Río Grande, Matías Romero, Oaxaca (n=4) 2015 | 1 | 1 | OUTC_species_2 | 1 | **OUTC_species_2** |
| *Paraneetroplus bulleri* (n=3) | Río Negro, Santa María Chimalapa, Oaxaca (n=2) 2014 | 1 | 1 | OUTC_species_3 | 1 | **OUTC_species_3** |
|  | Río Grande, Matías Romero, Oaxaca (n=1) 2015 | - | - | - |  |  |
| *Thorichthys maculipinnis* (n=2) | Río Grande, Matías Romero, Oaxaca (n=2) 2015 | 1 | 1 | OUTC_species_4 | 1 | OUTC_species_4 |
| *Vieja fenestrata* (n=2) | Río Grande, Matías Romero, Oaxaca (n=2) 2015 | 2 | 2 | OUTC_species_5  OMsCVII_species_8 | 2 | OUTC_species_5  **OMsCVII_species_8** |
| *Vieja zonata* (n=1) | Río Tequisistlán, Oaxaca (n=1) 2015 | 1 | 1 | OUTC_species_2 | 1 | **OUTC_species_2** |
| *Cichlasoma trimaculatum* (n=1) | Río Tequisistlán, Oaxaca (n=1) 2015 | - | - | - | - | - |
| *Maskaheros regani* (n=1) | Río Grande, Matías Romero, Oaxaca (n=1) 2015 | 1 | 1 | PBTC_species_2 | 1 | **PBTC_species_2** |
| *Dormitator maculatus* (n=11) | Tlacotalpan, Veracruz (n=6) 2014 | 6 | 2 | OBTC_species_4  *Ellipsomyxa papantla* sp. n. | 4 | **OUTC_species_1**  OBTC_species_4  **OMsCVII_species_5**  *Ellipsomyxa papantla* sp. n. |
|  | Tlacotalpan, Veracruz (n=5) 2015 | 4 | 4 | OUTC_species_1  OBTC_species_4  OMsCVII_species_5  *Ellipsomyxa papantla* sp. n. |  |  |
| *Gobiomorus dormitor* (n=2) | Río La Palma, Veracruz (n=2) 2014 | 1 | 1 | OBTC_species_5 | 1 | OBTC_species_5 |
| *Awaous banana* (n=3) | Río Negro, Santa María Chimalapa, Oaxaca (n=1) 2014 | 1 | 1 | *Myxidium zapotecus* sp. n. | 3 | ***Myxidium zapotecus* sp. n.**  ***Zschokkella guelaguetza* sp. n.**  OBTC_species_7 |
|  | Río Grande, Matías Romero, Oaxaca (n=2) 2015 | 3 | 3 | *Myxidium zapotecus* sp. n.  *Zschokkella guelaguetza* sp. n.  OBTC_species_7 |  |  |
| *Sarda sarda* (n=1) | Alvarado, Veracruz (n=1) 2014 | 4 | 2 | OUTC_species_1  PBTC_species_1 | 2 | **OUTC_species_1**  **PBTC_species_1** |
| *Rhamdia quelen* (n=6) | Río Negro, Santa María Chimalapa, Oaxaca (n=1) 2014 | 1 | 1 | OMsCVII_species_15 | 2 | OMsCVII_species_15  OMsCVII_species_16 |
|  | Río San Juan, Cristobal Obregón, Chiapas (n=5) 2014 | 1 | 1 | OMsCVII_species_16 |  |  |
| *Rhamdia guatemalensis* (n=4) | Río Grande, Matías Romero, Oaxaca (n=2) 2015 | - | - | - | 1 | OMsCVII_species_18 |
|  | Santa María, Guienagati, Oaxaca (n=1) 2015 | - | - | - |  |  |
|  | Catemaco, Veracruz (n=1) 2015 | 2 | 1 | OMsCVII_species_18 |  |  |
| *Synbranchus marmoratus* (n=2) | Río Negro, Santa María Chimalapa, Oaxaca (n=1) 2014 | 2 | 2 | OBTC_species_2  OBTC_species_3 | 2 | OBTC_species_2  OBTC_species_3 |
|  | Río Huixtla, Chiapas (n=1) 2014 | - | - | - |  |  |
| *Xiphophorus* sp. (n=1) | Río La Palma, Veracruz (n=1) 2014 | 1 | 1 | OMsCVII_species_6 | 1 | OMsCVII_species_6 |
| *Astyanax* sp. (n=3) | Río Negro, Santa María Chimalapa, Oaxaca (n=3) 2014 | 3 | 2 | *Myxidium zapotecus* sp. n.  *Zschokkella guelaguetza* sp. n. | 2 | ***Myxidium zapotecus* sp. n.**  ***Zschokkella guelaguetza* sp. n.** |
| *Eleotris* sp. (n=2) | Río La Palma, Veracruz (n=2) 2014 | 1 | 1 | OBTC_species_1 | 1 | OBTC_species_1 |
| *Paraneetroplus* sp. (n=2) | Río Negro, Santa María Chimalapa, Oaxaca (n=2) 2014 | 6 | 3 | OUTC_species_3  OBTC_species_6  OMsCVII_species_17 | 3 | **OUTC_species_3**  OBTC_species_6  **OMsCVII_species_17** |
| *Thorichthys* sp. (n=2) | Río Negro, Santa María Chimalapa, Oaxaca (n=2) 2014 | - | - | - | - | - |
