# Supplementary material for: Diversity of myxozoans (Cnidaria) infecting Neotropical fishes in southern Mexico
Source: Sci Rep. 2023 Jul 26;13:12106. doi: 10.1038/s41598-023-38482-2 (PMC10372099; doi:10.1038/s41598-023-38482-2)
Supplement: Supplementary file 6 — Supplementary Information 6. [file 41598_2023_38482_MOESM6_ESM.docx]

**Supplementary Data 6.-** Myxozoan prevalence for selected localities (n > 5 hosts collected), number of myxozoan species detected and proportion of myxozoan species detected by number of hosts collected.

| **Localities** | **Myxozoans prevalence (positive hosts/total number of hosts)** | **# Myxozoan spp.** | **# Myxozoans spp./# hosts collected** |
| --- | --- | --- | --- |
| Río Negro, Santa María Chimalapa, Oaxaca | 7/12 (58.3%) | 8 | 0.7 |
| Río Grande, Matías Romero, Oaxaca | 8/17 (47.1%) | 9 | 0.5 |
| Río La Palma, Veracruz | 11/20 (55%) | 13 | 0.7 |
| Tlacotalpan, Veracruz | 10/21 (47.6%) | 9 | 0.4 |
| Rio San Juan, Cristobal Obregón, Chiapas | 1/8 (12.5%) | 1 | 0.1 |
| Río Los Sabinos, Oaxaca | 3/7 (42.9%) | 2 | 0.3 |
| Río los Perros, Santa María, Oaxaca | 4/7 (57.1%) | 2 | 0.3 |
